# Supplementary material for: Reconciling kinetic and thermodynamic models of bacterial transcription
Source: PLoS Comput Biol. 2021 Jan 19;17(1):e1008572. doi: 10.1371/journal.pcbi.1008572 (PMC7845990; doi:10.1371/journal.pcbi.1008572)
Supplement: Attachment — Submitted filename: plos-cb-phillips-reconciling-kinetic-equilibrium-models-review.pdf [file pcbi.1008572.s002.pdf]

PCOMPBIOL-D-20-01024
Reconciling kinetic and equilibrium models of bacterial transcription
Morrison, Razo-Meija, Phillips

General comments

This paper is a substantial contribution to the senior author's longstanding programme of developing a predictive understanding of bacterial gene regulation. It examines a series of models for one of the simplest regulatory architectures, repressor competing with RNA polymerase at a single binding site, with the models differing in the level of coarse-graining of the RNA polymerase mechanism (see comment 1 below). There are three main contributions. First, for each model, algebraic formulas are determined for the mean and Fano factors of gene expression, in terms of the rate constants, from which the interesting finding emerges that, after suitable reparametrisation, the normalised mean levels of all the models become identical. The generating function method for undertaking the moment calculations is well known but the specific formulas which arise will be of interest to others in the field. Second, the models are confronted with single-cell mRNA FISH datasets acquired in a previous study, from which one model, in which RNA polymerase acts in bursts, emerges as consistent. Here, the careful use of Bayesian methods provides an attractive and systematic approach to the statistical extraction of the estimates. Third, the repressor binding and unbinding rates estimated for this bursty model are shown to broadly agree with previous studies, from which the authors conclude that the this model is able to accurately capture the kinetics of repressor-DNA interaction.

In summary, the paper offers further confirmation of the feasibility of predictive explanation for this "*hydrogen atom of regulatory biology*" (page 37). The main text is decidedly long and I can imagine that the discursive style, which adds to the length, may not be to every reader's taste. Personally, I find it engaging and useful because it draws the reader into a dialogue in which the reasoning and conceptual framework behind the results are clarified. This style of writing is rare in biology and serves a didactic purpose that can be beneficial to students and to those who are new to the field. I recommend publication of the paper but there are some issues detailed below which I would urge the authors to address first.

I believe that transparency improves peer review and therefore do not wish to remain anonymous, especially as I refer below to papers from my own lab.

Jeremy Gunawardena

Detailed comments

1. *Equilibrium versus non-equilibrium models*. This distinction is made throughout the paper, with "kinetic" sometimes replacing "non-equilibrium", as in the title. In particular, it is used to distinguish the models described in Figure 1B ("equilibrium models") and Figure 1C ("non-equilibrium models"). I feel this distinction is misleading for two reasons. First, it is inconsistent with the approach being taken for understanding energy expenditure in eukaryotic gene regulation, as described in several of our papers (PMIDs 27368104, 30762521, 31223115) and reviewed in PMID 32375018. Following Ptashne's "regulated recruitment" perspective, a distinction is made between "regulation", which is what happens prior to recruitment of RNAP, and "expression", which is what happens to RNAP after it is bound. (Evidently, this distinction is an abstraction which cannot be sustained indefinitely but it is widely used and useful currently.) From this perspective, regulation may or may not be at thermodynamic equilibrium,

while expression is always away from equilibrium. The key biological question is whether regulatory mechanisms are, or are not, operating away from equilibrium and, if they are, what functional consequences arise. In the eubacterial context, regulation, in this sense, seems to be largely at thermodynamic equilibrium and that is certainly the case for the present paper, in which repressor binds and unbinds from a single site. Accordingly, there is no biological significance to “non-equilibrium” behaviour in this context, other than the obvious fact that RNAP is operating, as it must, away from thermodynamic equilibrium. Second, the clear distinction between these models is that those in Figure 1B do not include the mRNA copy number within the states of the Markov process, while those in Figure 1C do include it. Taking this point a step further, what distinguishes these models is the level at which the mechanism of RNAP is coarse-grained (section 4.1.2 of PMID 32375018 may be helpful in seeing this point from a broader perspective). I strongly suggest that the authors adopt this coarse-graining distinction in place of the thermodynamic one which they have used. The results of this paper shed an important light on the process of coarse-graining but they do not, in my opinion, tell us much about “equilibrium” versus “non-equilibrium”.

The problems with this thermodynamic distinction are further revealed by the discussion of model 3 in Figure 1C on page 10. It is claimed that this model shows “*true non-equilibrium state-fluxes through promoter states*”. This assertion is problematic. The only reason the promoter states are away from equilibrium is because the transition between bound RNAP and paused RNAP is treated as irreversible. Irreversible transitions are always away from equilibrium but at the expense of an infinite entropy production. Had the transition been made reversible, as all transitions ultimately are, then the promoter states would still form a tree which continues to satisfy detailed balance (citation [45]). This is hardly a “*true non-equilibrium*”. For that to be plausible, there would have to be non-trivial reversible cycles in the regulatory part of the graph of the Markov process, which would thereby permit finite entropy production during regulation.

2. *Comments on King-Altman diagram method (pages 11, 13) and Hill's work (page 13)*. I was surprised to see these references. They are historically relevant but the authors seem to be unaware of the modern treatment based on the Matrix-Tree theorem (MTT) in the graph-based approach (citation [44]; for more discussion of Hill's contributions see Biddle et al PRE 101:062125 2020). Theorems of this kind go back to Kirchhoff but the version relevant here was first proved by Bill Tutte in 1948 and is a fundamental result in graph theory. King-Altman and Hill separately and independently found their own versions of this result, couched in ad-hoc languages (“diagrams”). These parochial formulations are divorced from their correct setting within mathematics, making it impossible to see the connections which link mathematics, physics and biology. The authors would be doing a service to the community by referring to the modern formulation and encouraging readers not to get stuck in the past.

   The authors make the point that these graph-theoretic methods may explain their calculations, especially the emergence of a “master curve” and the interpretation of its parameters (page 13). I agree with this assertion but the authors may wish to clarify the challenges involved. Specifically, the MTT applies, at present, only to finite graphs. In the context of the present paper, this means the models in Figure 1B, in which RNAP is not explicitly represented and mRNA number is not part of the state. The models in Figure 1C, in which mRNA number is represented within the state, require infinite graphs, for which an extension of the MTT remains an open problem (PMID 32375018), although this may not be such a limitation for determining the mean expression level.

3. *Using the graph-theoretic "linear framework" , as alluded to on page 13*. Since I have made this point previously to the senior author, I will not flog a dead horse here but simply draw attention to the several instances above, in which the graph-theoretic Markov process approach helps to clarify what the authors are trying to accomplish.
