# Supplementary material for: Reconciling kinetic and thermodynamic models of bacterial transcription
Source: PLoS Comput Biol. 2021 Jan 19;17(1):e1008572. doi: 10.1371/journal.pcbi.1008572 (PMC7845990; doi:10.1371/journal.pcbi.1008572)
Supplement: Attachment — Submitted filename: bursty_transcription_review.pdf [file pcbi.1008572.s004.pdf]

Reviews for: Reconciling Kinetic and Equilibrium Models of Bacterial Transcription

Muir Morrison[1], Manuel Razo-Mejia[2], and Rob Phillips[1, 2, *]

[1]*Department of Physics, California Institute of Technology, Pasadena, CA 91125, USA*
[2]*Division of Biology and Biological Engineering, California Institute of Technology, Pasadena, CA 91125, USA*
[*]*Correspondence:* `phillips@pboc.caltech.edu`

Author Summary

We are deeply grateful to the reviewers for such a thorough and thoughtful series of comments on our draft. As they discerned, this paper was the result of a pandemic-driven rush to a thesis defense date for the lead author. Paper submission was a prerequisite to even having the thesis defense and many of the elements of that thesis format were inherited by our imperfect submitted draft. We apologize to the reviewers that they were subjected to such a long manuscript. As a result of the comments, we have substantially rewritten the paper, relegating many of the details to the Supplement, seeking a more balanced review of the literature and attempting to explain the philosophy that drives our work which is quite different in spirit from some of the literature that might be favored by several of the reviewers. We hope that the resulting draft will be much more to their liking and now ready for publication. Thanks again to all of you for engaging so thoughtfully with our work.

1 PLoS Comp. Bio. 01

The authors present a detailed survey of simple biophysical models for the kinetics of transcription from a synthetic bacterial promoter. In particular, they highlight the challenges to discriminating between different models based on the available data of single-cell mRNA numbers. While the indistinguishability of models would be unsurprising (perhaps even self-evident) to practitioners of single-cell microbiology, the current work does an admirable job of rigorously demonstrating the problem and proposing some possible avenues towards addressing it. Thus, the manuscript is a welcome addition to the field.

Before publication is considered, however, a number of issues must be addressed. Those relate mainly to strong simplifying assumptions shared by all the models presented, assumptions that are made without a proper discussion of their validity. Making the cow spherical is often productive, but when doing so, the reader must be made aware of that, and the deviation from reality clearly stated. Specifically:

1) "the promoter is imagined to exist in a discrete set of states of occupancy, with each such state of occupancy accorded its own rate of transcription" (page 3). Is there direct experimental evidence for the existence of finite, discrete, activity states? It is easy to imagine how the known biology of bacterial transcription would violate that: For example, the supercoiling state of DNA is a continuous variable, which both modulates transcription and is affected by it (e.g., PMID: 31539491, PMID: 28707908). Its proper consideration would thus fall outside the premise of finite and discrete promoter states.

2) "assuming that the translation efficiency, i.e., the number of proteins translated per mRNA, is the same in both conditions" (page 5). This, too, appears violated by the known biology: The rates of transcription initiation, elongation, translation and degradation are tightly coupled (e.g., PMID: 20413502, PMID: 25964259, PMID: 31539491), hence the ratio of initiation rates is not expected a-priori to map linearly to protein concentration. Is there experimental evidence supporting this assumption? Note that the same argument also holds for measuring the total number of mRNA molecules per cell. To use mRNA number to deduce initiation rates requires showing a linear mapping between the two observables, despite the known coupling of mRNA initiation to elongation and degradation.

3) Another assumption shared by all models presented, but not stated explicitly, is that transcription propensity (per copy) does not change along the cell cycle. This seems especially dubious when modeling a promoter regulated by a repressor, for which it widely believed that transcription is tightly coupled to the passage of the replication fork (PMID: 8526893, PMID: 24562187, PMID: 31527794). How is this assumption justified?

We thank the reviewer for all of these comments and insights. First, we are in agreement that being up front about the assumptions is critical and we have attempted to remedy this shortcoming as explained below. It seems to us that in recent decades there have been a number of distinct approaches to come to terms with the complexity of biological processes such as transcription, each with their own merits. The approach we adopt here, in imitation of decades of work, much of which we cited, is thermodynamic models of promoter occupancy which explicitly coarse-grain away many effects and reduce problems to occupancy probabilities of discrete state spaces. We hold that it is important to subject these ubiquitous models to the scrutiny of a thorough theory-experiment dialogue and that is what we have tried to do. A second category of models as an alternative is mechanistically non-committal and instead posits many microscopically unspecified states and then seeks to search over the space of all such models for that one that best fits the data. That approach has its merits too, but is not at all what we are trying to do. The current paper attempts to carefully examine the former approach in the context of a specific regulatory architecture that we constructed explicitly with the purpose of making this kind of theory-experiment comparison.

To be more explicit about the specific points about inclusion of effects characterized by continuous rather than discrete variables, that is a fascinating point that we elaborate on a bit more here. Indeed, over the past several decades we spent a substantial effort in treating similar effects in the context of DNA looping where each and every looping configuration has its own free energy and contributes separately to the partition function [1], [2]. This step can be undertaken formally and when all the calculations are complete, one still has a set of discrete states to consider. For an example of our own use of these ideas (though many others have as well) see the equation in Fig. 1C of [1]. There are other classic examples in the biophysical literature where making such apparently unrealistic simplifications about discrete degrees of freedom are extremely effective. One such example is that of allostery. In principle allosteric molecules can exist in a continuous range of conformations, where the specific positions of all atoms in the molecule are to be specified. But coarse-graining this into two effective conformations has far reaching predictive power [3], [4], and here too one can rigorously integrate out degrees of freedom as shown in Equations 45-46 and 52 - 55 of [5]. In both the cases of DNA looping and allosteric induction we have shown explicitly and rigorously that one can literally integrate out these continuous degrees of freedom and we believe that ultimately, these kinds of insights are the point of building models and are a strong point as opposed to a bug. The objective of the paper is to bring the simple, mechanistic models with a finite set of discrete states that were first detailed by Ackers and Shea and many others in the time since, under careful experimental scrutiny. We adopt the philosophical mindset that one should start with the simplest description possible. If the predictions cannot be reconciled with the data, then, in an iterative process, we include more subtleties in the model. To that end, we added the following text to more explicitly explain our assumptions about discrete vs continuous states:

"... In both classes of model, the promoter is imagined to exist in a discrete set of states of occupancy, with each such state of occupancy accorded its own rate of transcription – including no transcription for many of these states. This discretization of a potentially continuous number of promoter states (due to effects such as supercoiling of DNA [6], [7] or DNA looping [1]) is analogous to how the Monod-Wyman-Changeux model of allostery

coarse-grains continuous molecule conformations into a finite number of states [4].”

We also thank the reviewer for pointing us to a series of very interesting references. Although the molecular details described in several of the papers could be included in a more complex model, we were unable to see how the assumptions we used violate any of the referenced effects. In our view, the critical assumption we make is to separate *in time* the repressor-bound state from the transcriptionally active states. We were unable to see a direct connection with the references provided by the reviewer since all the effects explored in the citations take place either during transcriptional initiation, or as the RNAP travels along the DNA, producing a transcript, though we agree that one should be up front about the fact that the occupancy-based models ignore these things from the outset.

We also thank the reviewer for the comment about gene copy number. We have definitely thought about the effect of gene copy number in the gene expression profile. Specifically in a recent publication [8], we accounted for cell cycle gene dosage dependence and found that it had a large effect on the cell-to-cell variability in *protein* copy number, but a surprisingly negligible impact on the variability in mRNA copy number.

Based on the three excellent points from the reviewer highlighted above, and other suggestions from other reviewers, we added the following paragraph to the Discussion to call attention to the wide variety of assumptions that are behind the models considered here:

“It is important to be clear on the nature of the assumptions present in all models, including model 5 from Figure 2(A). First, as with many of the models used routinely in the analysis of transcription, the models considered here imagine the promoter as existing in a series of discrete states. This assumption is clearly an oversimplification due to effects such as DNA supercoiling [6], [7] or DNA looping [1] which are both parameterized by continuous variables describing DNA shape; although there are formal and very interesting ways of integrating out such degrees of freedom. Another facet of the models used here is that they can feature irreversible processes characterized by only a single rate process with no corresponding return pathway at odds with thermodynamics. Such models are mathematically convenient and serve only as an approximation to the more realistic situation in which the forward rates are much larger than the backward rates. One consequence is that the inferred rates for the transcription burst rate and burst size can only be thought of as effective rate constants. Another idealization of the models considered here is that when computing the fold-change in gene expression (Section 2) we assume that the translation efficiency of an mRNA transcript is the same in cells with and without regulation. There might be some known biological effects that could limit the applicability of this assumption such as cooperation between the transcription and translation machinery [9], or coupling of mRNA transcription and degradation [10] among other potential effects. Another implicit assumption in all of the models discussed here is that the effects due to changes in gene copy number during the cell cycle have no effects on the expression profile. For moderate growth rates such as those used in [11] from which we obtained the data, at all points during the cell cycle there are at most two copies of the gene of interest. In a recent publication, we explored the effects that this change in gene dosage can have in gene expression, finding that the effects are much more drastic at the protein level [8].”

Additional matters:
4) "disproving the universal noise curve from So et. al. [59]" (page 18). How was the universal noise curve disproved? Universal noise/mean relations, similar to those reported in ref #59, were repeatedly observed, in both bacteria and eukaryotes (see, e.g., PMID: 24311680, PMID: 25858977). Moreover, later reports showed that the trend is observed even when accounting for gene dosage effects (PMID: 26965629). Thus, the statement above requires clarification.

We thank the reviewer for this comment. Although the claims from So et al [12] are very interesting and suggestive, the data presented in Jones et al. [11] are clearly inconsistent with a universal curve as seen in Figure R1. We are unsure how else to interpret the results of Figure R1(B) other than the experiments they report are wrong, or, that they demonstrate unequivocally that depending upon the nature of the regulatory architecture, the noise will take different forms (and is thus not universal). We are strongly in favor of the latter hypothesis, especially given that the theory curves were plotted before the data was taken and the reentrant behavior is exactly what is expected in theoretical models. In particular, the different curves in Figure R1(B) of Fano factor vs mean mRNA copy number have different maxima depending on the strength of the promoter. This observation is not consistent with the proposed universal curve.

Although we believe in our data and the claim, we removed the phrase from the main text in order to avoid the potential controversy that might still exist in the field.

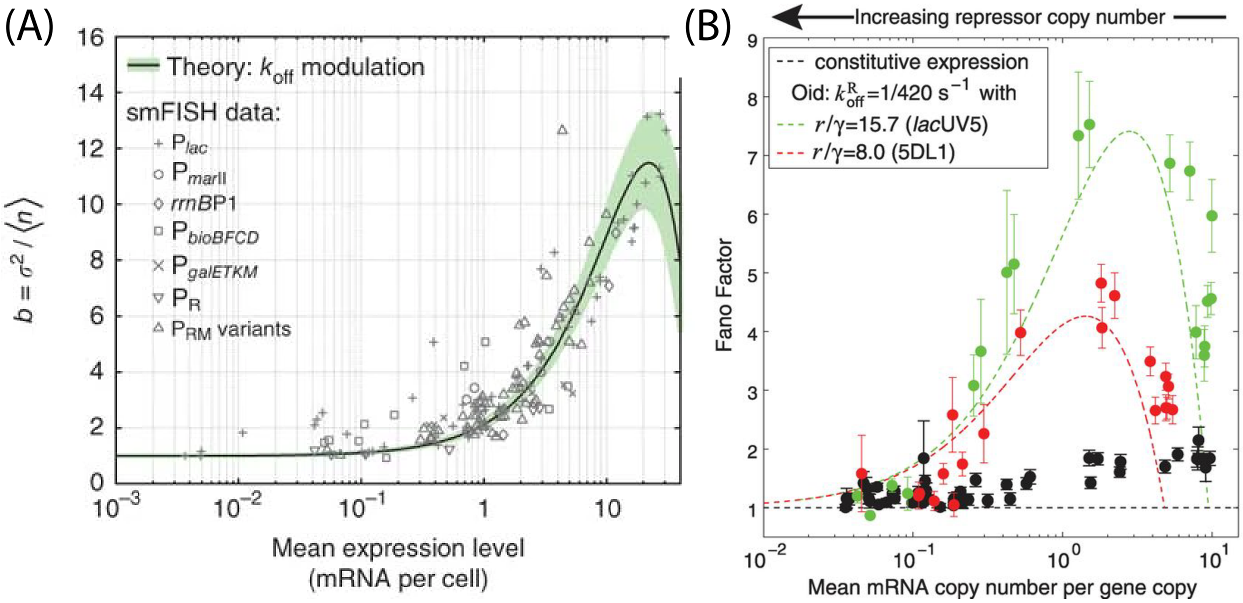

Figure R1. Different experimental examinations of the nature of gene expression noise (A) "Universal noise curve" shown in [12]. In this work the claim is that, regardless of the regulatory architecture of a bacterial promoter, the noise (standard deviation / mean) in mRNA counts scales exactly the same as a function of the mean mRNA count. (B) Equivalent plot as in (A) from [11], but with the difference that all data was taken from a single genetic architecture (simple repression). Dashed lines were drawn without fitting anything to the shown data.

5) The manuscript is too long. It reads more like a monograph or a student's thesis (and a good one, as such) rather than a journal research paper. It is up to the editor, but I would advise that some of the specific models be moved to the supplementary material, to create a more compact text.

We are grateful to this and the other reviewers that highlighted this point. As noted in our summary at the beginning and as this reviewer guessed, indeed, the lead author was under strict pandemic time pressure to have this paper submitted as part of completing his PhD at Caltech and we were enthusiastic about the long-format arguments that had been made there, but agree that the paper needed a restructuring where most of the calculations were sent to the supplementary material. For this resubmission we have significantly shortened the manuscript, directing the interested readers to the different appendices for further mathematical details. We have also moved all intermediary discussion sections to the end of the paper.

2 PLoS Comp. Bio. 02

This manuscript examines a few simple linear models of state dependent gene expression used to describe the regulation of gene transcription under control of varying levels of a repressor. The manuscript begins by examining the equilibrium mean level of gene expression, which boils down to expected value of the transcription rate (over the probability mass for the different states) divided by the degradation rate. The authors show that the mean fold change (expression with 'R' repressor concentration relative to expression in the absence of the repressor) versus 'R' collapses to a simple monotonic function parametrized by (i) an effective free energy term that depends on 'R' and (ii) a constant 'rho' that depends on the model structure, but not on 'R' itself. For non-equilibrium models, a similar form is derived, but where the dependence on 'R' is lumped into rate constants k_R^+ and k_R^- for repressor binding/unbinding (presumably with k_R^+ being linearly dependent on the repressor concentration). Next, the manuscript examines the steady state variance predicted by their models, again using a very simple and well-studied class of models for which all propensity functions are linear and where there are no mechanisms of feedback. Then, the manuscript gets into more interesting work with Bayesian parameter inference for the parameters of a simple bursting kinetics model from single-molecule RNA FISH data. They examine published data (from the same group) with nine different combinations of repressor binding sites and repressor concentrations, and they estimate uncertainties in the shared parameters among these nine conditions. The authors argue that this analysis of the previous data confirms the validity of their equilibrium models.

The manuscript addresses an interesting topic of how to connect different types of gene expression models and what mechanistic properties can be inferred from single-cell RNA FISH data, which are both important topics in the single-cell research community. The Bayesian analysis of a single model to describe multiple different combinations of promoters and repressor concentrations is a very nice example for how simple models can be effectively integrated with single-cell data. The results and approaches appear to be correct. Unfortunately, much of the presented model development and analyses (especially sections 2 and 3) was wasted rehashing several extremely well-studied models, so that it became very difficult to discern what new insight is being provided by the authors, and what is a simple restating of known results. This issue was compounded by the fact that the manuscript does not provide adequate citations to existing research in the field, especially given the vast amount of recent work that has been performed on the identification or, and uncertainty quantification for, gene regulation models using single-cell RNA FISH data. Finally, the manuscript makes a big deal about how the authors' analyses confirm the validity of their equilibrium model, but (1) at least as far as I could find, the authors provide no convincing statistical evidence that supports this claim while rejecting some competing null model and (2) the actual importance or novelty of this claim is not clear. Overall, as written, I expect this manuscript will have a low to moderate impact among the general readership of PLoS Computational Biology, and it might be more appropriate for a more specialized physics or physical biology journal.

Major Comments:
1) The authors first result (Sec. 2, the collapse of the fold change curves for the presented simple linear models) is not surprising for either the equilibrium or non-equilibrium models. Each model described in the first part of the manuscript can be split into two groups of states: (i) a single repressor-bound state and (ii) a bunch of interconnected unbound states. At least for the models described in Fig 1, the authors are aware that there is only a single path to or from the repressor bound state, which guarantees detailed balance between these two groups of states (this would still be true even if the studied models were extended to include multiple different bound repressor states). As such, the conditional steady state probability mass within each group of states will be identical with or without the single repressor state, and this will enable the form shown in Figure 1D — although with multiple bound repressor states, the form for ΔF_R could be more complicated with its form dictated by the interactions between the states in that group. Given that the authors only consider one model for the states within the repressor group (i.e., a single bound state), it is not clear what we learn by the observation that all these models have the same functional form for the fold change.

We thank the reviewer for this thoughtful series of comments. At the outset, it seems we might have some differences of philosophical perspective that are worth mentioning in the hope that we might agree that there is room for multiple perspectives to catapult the field forward. First, both for us and the reviewer (thanks for identifying yourself Brian, we greatly appreciate it), the question of rigorous, reproducible knowledge is vitally important to the scientific enterprise. Further, we were not trying to write a surprising paper, nor do we even think that "surprise" should be accorded much weight in deciding on the value of scientific research. We also are of the mind that what is surprising and what is not surprising is a subjective matter. Our objectives here were to dig into a class of well known models going back to Ackers and Shea, developed beautifully by Buchler, Gerland and Hwa, practiced by many in both bacterial and eukaryotic transcription and popularized repeatedly by Ptashne that are sometimes known as "regulated recruitment" models or more appropriately, as occupancy models. These models are ubiquitous, used in contexts ranging from bacterial transcription (including a beautiful paper by reviewer 4 on MarA) to yeast (see Erin O'Shea, Barak Cohen and others on Pho5) to Eran Segal with Ulrike Gaul, John Reinitz and many others in flies. These models are in a different category than those considered by Neuert/Munsky *et al.* in that they posit a specific constellation of transcription factor binding sites and then work out the consequences. Our aim here was to work within that limited class of models and really try to rigorously examine the extant models and see how well they do. Experimentally, we specifically designed the promoter architecture to have a very precise arrangement so that it was clear which competitor models to consider and thus although the reviewer makes it sound as though the right move would have been to vastly expand the class of models under consideration, to our way of thinking, that would have missed the point of the whole study.

We think we have a pretty good command of the literature in bacterial transcription and are

puzzled by the idea that data collapse is a commonplace in this field. On the subject of data collapse, to the best of our knowledge, Ned Wingreen in the context of bacterial chemotaxis and quorum sensing and our own work in several papers is it. We admire and are intrigued by the kinds of models found for example in Neuert/Munsky et al., but view them as a different approach with different objectives. Thus, we don't know where to go with Major Comment 1. If the reviewer thinks that this kind of result has been derived theoretically and then put to the test experimentally, we would definitely like to know what paper we should be citing to illustrate those points. Obviously, this would take some of the wind out of our sails, but we are hard pressed to believe that this is all well known. Further, the reviewer makes the comment "what we learn" –we push back in the same way that he did– the literature is replete with these kinds of models. We are trying to examine the extent to which those specific models (not huge swathes of model space) have to say about data on transcription. Part of "learning" in science has to do with just carefully and rigorously examining topics from A-Z and there should be room for this kind of thing in addition to the intense (and in our opinion unjustified focus on "surprise"). As far as we know this particular result has not been previously shown to this extent in the gene expression modeling literature. We therefore think that the contribution –with the specific insight correctly listed here by the reviewer– can benefit the community by highlighting the impossibility to discern different models at the level of mean gene expression. So one goal of this work is to provide a rigorous and thorough treatment of this result with careful derivations and comparisons of the relevant plausible models. For many workers in the field, we would argue that these things are not obvious since all of us in talks and in other papers have encountered outright rejection of the idea that equilibrium ideas (i.e., separation of time scales) has any place in describing biological systems. Here the careful description of the equivalence of the functional forms of these two approaches is a useful step forward, and again, we are unaware of the citations that the reviewer might have in mind where this has been previously demonstrated.

As the reviewer correctly observes, with additional repressor binding sites, or non-exclusive binding of repressor and RNAP, the equivalence of thermodynamic and kinetic models is no longer guaranteed. We did not consider such models in this work since the mRNA FISH dataset we were using only contained constructs with exclusive RNAP and repressor binding sites. And, we reiterate, this is not an appeal to the data from some other laboratory. We designed these strains precisely for the purposes of isolating this specific and simple architecture to see how well we all really understand it. We are of the mind that if we fail in making sense of this simplest of regulatory architectures, that makes the analysis of more complex architectures (which we have also considered) even more suspect. Indeed, that is the whole point of constructing the strains that were used in these experiments in the first place, namely, to insulate the system from a myriad of other biologically important factors. We are of the mind that biological enquiry would benefit from more examples like this of taking the full biological system and repurposing it through simplification to make a direct theory-experiment comparison possible. And from the theoretical side, the proliferation of new parameters makes it difficult to reach any general conclusions without data to constrain the parameters. Even if we had such data, the Bayesian inference problem would be harder than what we do here since a closed form likelihood would likely be unavailable for these more complicated models. So the generalization to these models seemed to us best left for a

separate study that had the corresponding carefully designed strains to insulate that system like we did here. We have added the following red text to the discussion:

“ Our key insights can be summarized as follows. First, as shown in Figure 1, the mean expression in the simple repression architecture is captured by a master curve in which the action of repressor and the details of the RNAP interaction with the promoter appear separately and additively in an effective free energy. Interestingly, as has been shown elsewhere in the context of the Monod-Wyman-Changeux model, these kinds of coarse-graining results are an exact mathematical result and do not constitute hopeful approximations or biological naivete [13], [14]. The fact that thermodynamic and kinetic models for the simple repression motif can not only be reconciled, but are indistinguishable from each other at the level of mean mRNA count is a consequence of the possible transitions between promoter microstates. The promoter microstates in all models shown in Figure 1 can explicitly be separated into two groups: 1) promoters with repressor bound, and 2) all other promoters states. The single path between these two groups of states guarantees detailed-balance between the groups. We can therefore separate *expression* related transitions, which by definition must be out of equilibrium, with *regulation* transitions that can in principle be in equilibrium. This implies that the probability distribution of the promoter microstates not involving the repressor take the same functional form, allowing us to write the fold-change as an effective free energy involving a regulation term, ΔF_R, and a promoter details term, ρ. ”

We have also shortened the manuscript (especially Section 2), sending most of the mathematical details to the supplemental materials. We still list the general result of this section as something that the reader might find interesting or even surprising, depending on their background, but direct the interested readers to the Appendix to get all details on the derivations.

> 2) In the second part of the manuscript (Sec 3), the authors extend their analysis to explore the variance, and Fano factors, under various simple linear models with multiple expression states. Like all models with linear propensity functions, the first and second moments for these models are known (not just for their steady state values as presented in this manuscript, but also for their temporal correlations and transient responses). The bursting model at the end of section 3 is also well studied in the literature. It was difficult for me to connect the insight from section 2 to that of section 3? Is there any relevance of the fold change functional form from Section 2 when examined through the lens of Fano factors or distributions? Or are these meant to be two unconnected analyses?

We again thank the reviewer for this comment. Based on this and the previous comment, we have attempted to clarify the logical progression here. The reviewer is correct that all these models have been studied previously in the literature, as the citations in Figure 2 and in-text provide examples of; indeed, part of our point here is precisely to uncover hidden connections between existing models. But we want to reiterate, this paper did not claim to introduce some new model. We have been on a decade long effort to construct a rigorous

theory-experiment dialogue using a specific set of regulatory architectures and a minimal parameter set, and the point here is to examine the kinds of models that have been invoked and to explore several of the key measurables favored in the literature.

In short, there is no direct relevance between the fold-change functional forms and the Fano factors or distributions, though we think it is very important to remember what people measure. Going back to the very important work of Oehler et al., but many other contributions as well, the fold-change has been one of the primary tools used in the context of bulk transcriptional measurements. As such, it is of great interest. Because of the advent of fluorescent reporters, fluorescence microscopy, flow cytometers, etc., there has been a similar explosion of measurements of different versions of the second moment. As a result, we wanted to be clear on what the different models have to say about these measurables, in addition to the very interesting question of the full distributions themselves. The punchline of Section 2 (highlighted in the last paragraph of the section) is that all these models are indistinguishable at the level of means. Following from that, a natural concern that arises is whether these models are just mathematical tautologies or whether the fitted parameters in the models (i.e., binding energies and rate constants) correspond to the real physical concepts we have ascribed to them. Section 4 directly addresses that question with Bayesian inference on mRNA FISH data. We could have simply done the hard work of Bayesian inference on *all* the models, but we realized that several of the models could be ruled out with much less effort, simply by comparing their Fano factors to the data. So we made a detour in Section 3 to do this first, motivating the reduced set of models that we analyze in Section 4. Our intent was to create a self-contained treatment of all the models. But in hindsight, since most of the results of Section 3 either already existed in the literature or are straightforward generalizations of them, we have shifted most of the details to the supplementary material.

> 3) The use of Bayesian analysis and MCMC to infer parameters from single-cell RNA FISH data is a little more interesting. Although this kind of analysis has now also been done many times in the recent literature, the authors' application of the MCMC to estimate the posterior parameters under many different promoters (Fig. 3C) was interesting. Again, it is not clear how this section relates back to the Section 2. Perhaps this was meant to be addressed in Fig. 3(D), but I could only find one sentence in the manuscript about this panel (page 29) and it did not provide much insight. Overall, if the focus of the paper could have been on this analysis of multiple data sets, perhaps the paper would have been more compelling to me.

We are thankful with the reviewer for this comment. Perhaps it is important to differentiate the philosophical approach in this particular manuscript compared with other very influential papers on gene expression, especially because at this point our perspectives differ on what is "interesting" and we are hopeful that the reviewer will acknowledge that there are people in the community (such as Ido Golding, Al Sanchez, Barak Cohen and many others) that find the rigorous underpinnings of thermodynamic models to be interesting in and of themselves. A lot of the exceptional literature on bursty gene expression –including some of the reviewer's own very interesting work [15], [16]– takes a fundamentally different approach to ours in terms

of the role that theoretical models play. Specifically, the reason we chose to model arguably the simplest genetic circuit possible (one binding site for a single transcriptional repressor) is because we want to write down a model that, although coarse-grained to a big extent, still speaks to what we think is the physical reality of the process of transcription and for which the ubiquitous notion of "occupancy" is entirely clear. If we had taken any other genetic architecture such as multiple binding sites for the same transcription factor, or an activator rather than a repressor, we would not consider the same models discussed throughout this manuscript.

What we bring to the table is a rather more mechanistic view of what the models are supposed to represent. One could argue that in the models we explore there is still a huge degree of simplification, and that the way we treat the phenomenological rates of the unregulated promoter is not very different from just taking the commonly used two-state "telegraph" model and fitting it to the data. But we argue that the result of finding the inferred rates agree with the binding free energy obtained with a completely orthogonal method, would have only been possible given the approach we took in which models are coarse-grained to the point where there is still some physical reality to what the kinetic rates mean.

We are not aware of any other manuscript in the literature that takes a similar approach. If the reviewer knows of some paper that we missed out in the literature where such a rigorous comparison between the two theoretical formalisms was done, we would love to know about it.

> 4) The discussion in Sections 4.2.2 and 4.2.3 need to be fleshed out a little more — as written, these sections are very confusing, and it is not clear how one should evaluate the statistical significance of the authors' claim that this is a "striking confirmation of the validity of the equilibrium models (page 34)" . Can the authors introduce and compare to some sort of null model or provide some simulated data to demonstrate that if their theory of equilibrium models and repressor binding energies were incorrect this analysis would have provided a clear rejection of that theory? Can the authors give a p-value for the term 'striking'?

We thank the reviewer for this comment. We apologize if the use of the word striking was taken as inappropriate. It is hard to avoid the impression that the reviewer has some real annoyance with us both scientifically and in terms of our presentation. What we meant by this point is the following: On the one hand, we have tried really hard to "disprove" thermodynamic models of gene regulation. From testing if the model breaks down when mutations are introduced to the transcription factor itself [14], to changing the physiological conditions in which the cells are grown [17], this class of models has proven over and over again to be an extremely robust description of the mean gene expression level. On the other hand, the fact that the repressor kinetics left a signature in the full mRNA distribution that allowed us to infer the kinetic rates, and these agree with the thermodynamic model, we think is striking. There is no reason this needed to be the case. The inference of the repressor rates involves a convoluted two-state model with a non-intuitive steady-state distribution that involves confluent hypergeometric functions. But still, from this theory-data comparison

that looks at entire distributions rather than means, using a different language (chemical master equations vs. equilibrium statistical mechanics) we find agreement at the level of the inferred binding free energy.

> 5) After the very nice parameter estimation shown in Fig. 4(A), I was expecting the model to be tested on its ability to predict the distributions for the held out cases (e.g., Oid at 0.5 or 10 ng/ml or O1 at 0.5 ng/ml) using the parameters inferred from the other promoter/repressor conditions. These predictions would have made for a much more compelling version of Fig. 4(C). As presented, Fig 4(C) only seems to show plots for data that were already used in the fitting.

We agree with the reviewer that such predictions would have been a nice addition to the work presented here. But in practice we were not able to perform such comparisons. We found that fitting a single operator/aTc concentration at a time with a single binding and unbinding rate did not yield a stable inference for most of the possible operator/aTc combinations. In other words, a single dataset could not independently resolve the binding and unbinding rates, only their ratio as set by the mean fold-change in Figure 1 in the main text. Only by making the assumption of a single unique binding rate for each repressor copy number and a single unique unbinding rate for each binding site, as done in Figure 4(A), was it possible to independently resolve the rates and not merely their ratios.

We also note that we found it necessary to exclude the very weakly and very strongly repressed datasets from Jones et. al. [11]. In both cases there was, in a sense, not enough information in the distributions for our inference algorithm to extract, and their inclusion simply caused problems for the MCMC sampler without yielding any new insight. For the strongly repressed data (Oid, 10 ng/mL aTc), with $> 95\%$ of cells with zero mRNA, there was quite literally very little data from which to infer rates. And the weakly repressed data, all with the repressor binding site O3, had an unbinding rate so fast that the sampler essentially sampled from the prior; the likelihood had negligible influence, meaning the data was not informing the sampler in any meaningful way, so no inference was possible.

This discussion is part of our supplementary material, but we have also added the following details to the discussion in the main text:

"With the results of the constitutive promoter in hand, we then fix the parameters associated with this class of promoters and use them as input for evaluating the noise in gene expression for the simple repression motif itself. This allows us to provide a single overarching analysis of both the constitutive and simple repression architectures using one simple model and corresponding set of self-consistent parameters, demonstrating not only a predictive framework, but also reconciling the thermodynamic and kinetic views of the same simple repression constructs. More specifically, we obtained values for the transcription factor association and dissociation rates by performing Bayesian inference on the full mRNA distribution for data obtained from simple-repression promoters with varying number of transcription factors per cell and affinity of such transcription factors for the binding site. The free energy value obtained from these kinetic rates –computed as the log ratio of the rates– agrees with previous inferences performed only from mean gene expression measurements, that assumed a ther-

modynamic rather than a kinetic framework [13], [18]. Interestingly, to constrain the binding and unbinding rates inferred for Figure 4, all of the rates need to be simultaneously fit. This is because a single combination of operator (repressor binding site) and aTc concentration (repressor copy number) cannot constrain both rates independently, but only their ratio (See Appendix 3 for further details)."

> 6) The manuscript is missing important citations to key work in this research domain. Admittedly, there are hundreds (if not thousands) of theoretical and experimental papers that invoke the bursting gene expression models, a comprehensive list would be impossible. But since the authors saw fit to cite R. Phillips' work at least 18 times, they could have made a little more effort to capture the other relevant work on the topic. Some particularly relevant articles would include work by Hana El Samad, Ramon Grima, Srividya Iyer-Biswas, Michal Komorowski, Mustafa Khammash, Heinz Koeppl, Andrew Mugler, Brian Munsky, Arjun Raj, Jakub Reuss, Abhyudai Singh, Eduardo Sontag, Michael Stumpf, and Alexander van Oudenaarden, to list just a handful of research labs that have presented extensive analyses of bursting gene expression models, have fit these models to single-cell data (including RNA FISH data), have quantified parameter uncertainties using MCMC analyses, and more.

Of course, we share the reviewer's commitment to veracity in citing the work of others, and are grateful for the opportunity to improve our referencing. This is one of the inheritances of extracting this paper from a rushed PhD thesis defense for which we apologize profusely. Further, we are great admirers of many of these people, including those reviewers that identified themselves. Indeed, with Hernan Garcia, one of us is trying to write a definite and thorough treatise on transcription and many of these people, and others such as Eran Segal, Barak Cohen, Angela de Pace, Tom Kuhlman, Ido Golding, Jordi Garcia-Ojalvo, Justin Kinney, and others have been advising us and we hope to get the advice of the reviewer himself.

That said, our strategy on citations here is to specifically address a very precise issue, namely, the dialogue between theory and experiment, aimed at using one minimal and self-consistent parameter set that applies to different experimental methods. In references [13], [19]–[21] we cited a long list of works on thermodynamic models and in references [8], [11], [22] we made a corresponding list on chemical master equations.

We are slightly puzzled by the reviewer's concept which appears to aim at listing people who have worked on transcription. We have enlarged the two lists cited above on thermodynamic models and chemical master equations to give a more complete list of those who have done important work on these problems. This paper advances no claim of novelty in terms of studying bursting theoretically or experimentally. The claimed novelty is the attempt to explicitly compare a myriad of models and corresponding data. If this reviewer knows of a paper that already did what we are doing here, we would love to get the proper citations.

Minor Comments.
7) Please introduce the variable 'R" and other necessary notation before the first reference to Figure 1. It is confusing to use both β and k_bT in the figure. Please choose one or the other. Similarly, the variable N_{NS} is used in Fig 1 too long before it is defined under Eqn 4. This is confusing.

We thank the reviewer for this comment. We now introduce the variable definitions in early in Section 2. Specifically we now have in the second paragraph of the section:

"'The first class of models that are the focus of the present section on predicting the mean level of gene expression, sometimes known as thermodynamic models, invoke the tools of equilibrium statistical mechanics to compute the probabilities of the promoter microstates [19], [23]–[31]. As seen in Figure 1(B), even within the class of thermodynamic models, we can make different commitments about the underlying microscopic states of the promoter. Model 1 considers only two states: a state in which a repressor (with copy number R) binds to an operator and a transcriptionally active state. The free energy difference between the repressor binding the operator, i.e. a specific binding site, and one of the N_{NS} non-specific sites is given by $\Delta\varepsilon_R$ (given in k_BT units with $\beta \equiv (k_BT)^{-1}$). Model 2 expands this model to include an empty promoter where no transcription occurs, as well as a state in which one of the P RNAPs binds to the promoter with binding energy $\Delta\varepsilon_P$. Indeed, the list of options considered here does not at all exhaust the suite of different microscopic states we can assign to the promoter."'

8) In Fig. 4C, the use of ECDF is nice for distributions whose support is well spread over the positive integer numbers (e.g., for the low repressor concentrations), but this visualization can hide deviations for distributions whose support is mostly at zero (e.g., for high repressor concentrations). It would be useful for the authors to show the histograms and probability mass vectors as well as the ECDFs. The authors are aware of these differences, and it is much appreciated that they discuss them in Section 4.2.4, but I worry that these 'oddities' or 'minor quibbles' are inadvertently diminished by the choice to use ECDFs instead of probability density histograms.

We thank the reviewer for raising these concerns. The use of ECDF in our theory-experiment comparison for Figure 4(C) allowed us to show multiple distributions in the same plot. We agree that showing a histogram version of the plot can be useful for the readers to make a deeper judgment of the quality of our predictions, so we added Figure R2 to the Supplementary material.

9) Typo on page 32: "for a given inducer concentrations." Remove 's' from 'concentrations'

We are thankful for the careful reading from the reviewer. This typo has been fixed.

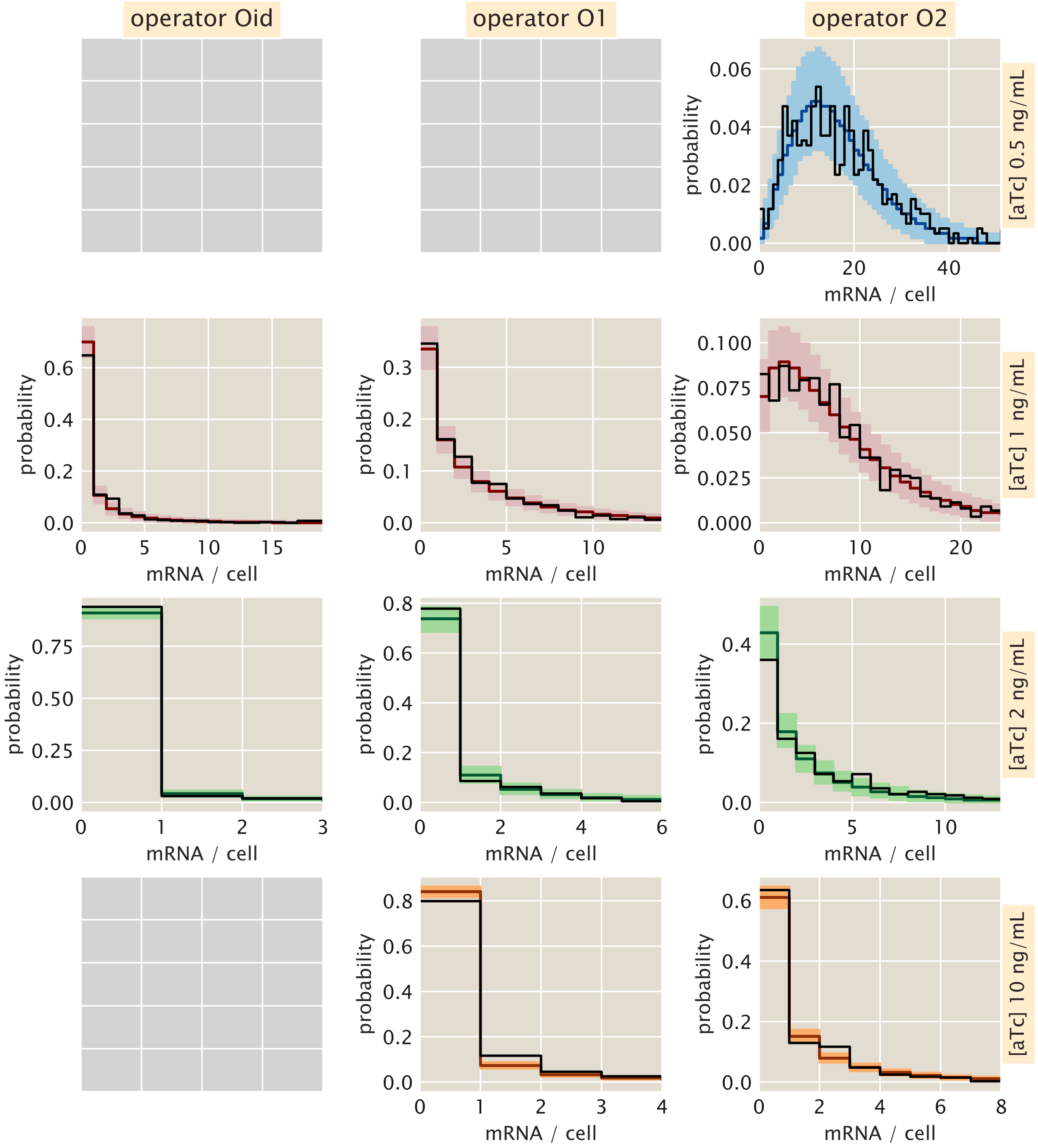

Figure R2. Theory-experiment comparision of mRNA distributions for regulated promoters. Comparison of the inference (color shaded area encloses the 95% of all posterior predictive check samples while the color solid line represents the median of all samples) vs the experimental measurements (black lines) for different regulated promoters with different operators (columns) and aTc concentrations (rows) from Ref. [11].

10) While this may be a matter of personal taste, I found the informal conversational tone of the manuscript (e.g., words like ‘beautiful’, ‘bonanza’, ’quibble’, etc) to be distracting. Such flowery language is great for a short perspectives article or opinion piece, but for a manuscript of this length, rigor is key and imprecise language becomes annoying. It would be nice if the authors could tighten up their message and try to present a more concise description of their key findings.

We sincerely apologize for the use of such language. Part of the text was lifted from a PhD thesis that carried this informal tone. In this reviewed manuscript we have now removed this kind of language.

3 PLoS Comp. Bio. 03

This paper is a substantial contribution to the senior author's longstanding programme of developing a predictive understanding of bacterial gene regulation. It examines a series of models for one of the simplest regulatory architectures, repressor competing with RNA polymerase at a single binding site, with the models differing in the level of coarse-graining of the RNA polymerase mechanism (see comment 1 below). There are three main contributions. First, for each model, algebraic formulas are determined for the mean and Fano factors of gene expression, in terms of the rate constants, from which the interesting finding emerges that, after suitable reparametrisation, the normalised mean levels of all the models become identical. The generating function method for undertaking the moment calculations is well known but the specific formulas which arise will be of interest to others in the field. Second, the models are confronted with single-cell mRNA FISH datasets acquired in a previous study, from which one model, in which RNA polymerase acts in bursts, emerges as consistent. Here, the careful use of Bayesian methods provides an attractive and systematic approach to the statistical extraction of the estimates. Third, the repressor binding and unbinding rates estimated for this bursty model are shown to broadly agree with previous studies, from which the authors conclude that the this model is able to accurately capture the kinetics of repressor-DNA interaction.

In summary, the paper offers further confirmation of the feasibility of predictive explanation for this "hydrogen atom of regulatory biology" (page 37). The main text is decidedly long and I can imagine that the discursive style, which adds to the length, may not be to every reader's taste. Personally, I find it engaging and useful because it draws the reader into a dialogue in which the reasoning and conceptual framework behind the results are clarified. This style of writing is rare in biology and serves a didactic purpose that can be beneficial to students and to those who are new to the field. I recommend publication of the paper but there are some issues detailed below which I would urge the authors to address first. I believe that transparency improves peer review and therefore do not wish to remain anonymous, especially as I refer below to papers from my own lab.

Jeremy Gunawardena

1) Equilibrium versus non-equilibrium models. This distinction is made throughout the paper, with “kinetic” sometimes replacing “non-equilibrium”, as in the title. In particular, it is used to distinguish the models described in Figure 1B (“equilibrium models”) and Figure 1C (“non- equilibrium models”). I feel this distinction is misleading for two reasons. First, it is inconsistent with the approach being taken for understanding energy expenditure in eukaryotic gene regulation, as described in several of our papers (PMIDs 27368104, 30762521, 31223115) and reviewed in PMID 32375018. Following Ptashne’s “regulated recruitment” perspective, a distinction is made between “regulation”, which is what happens prior to recruitment of RNAP, and “expression”, which is what happens to RNAP after it is bound. (Evidently, this distinction is an abstraction which cannot be sustained indefinitely but it is widely used and useful currently.) From this perspective, regulation may or may not be at thermodynamic equilibrium, while expression is always away from equilibrium. The key biological question is whether regulatory mechanisms are, or are not, operating away from equilibrium and, if they are, what functional consequences arise. In the eubacterial context, regulation, in this sense, seems to be largely at thermodynamic equilibrium and that is certainly the case for the present paper, in which repressor binds and unbinds from a single site. Accordingly, there is no biological significance to “non-equilibrium” behavior in this context, other than the obvious fact that RNAP is operating, as it must, away from thermodynamic equilibrium. Second, the clear distinction between these models is that those in Figure 1B do not include the mRNA copy number within the states of the Markov process, while those in Figure 1C do include it. Taking this point a step further, what distinguishes these models is the level at which the mechanism of RNAP is coarse-grained (section 4.1.2 of PMID 32375018 may be helpful in seeing this point from a broader perspective). I strongly suggest that the authors adopt this coarse-graining distinction in place of the thermodynamic one which they have used. The results of this paper shed an important light on the process of coarse-graining but they do not, in my opinion, tell us much about “equilibrium” versus “non-equilibrium”.

We thank the reviewer for this incredibly insightful and thoughtful comment. We are also grateful to this reviewer for his continued patience as we try to find a common language and understanding with him on how best to talk about and test our thinking on modeling transcription in prokaryotes and eukaryotes alike. We completely agree with the reviewer’s view on how our paper speaks to the different modeling techniques that coarse grain at different levels the molecular events involving the RNAP. As the reviewer will find throughout the paper we changed all references of “*equilibrium*” and “*non-equilibrium*” to “*thermodynamic*” and “*kinetic*,” respectively. Furthermore, although the structure of the paper has changed drastically from the previous submission based on all reviewers’ comments, and pointing to direct changes from the previous version is difficult, here is a relevant paragraph in Section 2 that touches on the excellent points brought up by the reviewer:

“The second class of models that allow us to access the mean gene expression use chemical master equations to compute the probabilities of the different microscopic states [32]–[39].

The main differences between both modeling approaches can be summarized as: 1) Although for both classes of models the steps involving transcriptional events are assumed to be strictly irreversible, thermodynamic models force the regulation, i.e., the control over the expression exerted by the repressor, to be in equilibrium. This does not need to be the case for kinetic models. 2) Thermodynamic models ignore the mRNA count from the state of the Markov process, while kinetic models keep track of both the promoter state and the mRNA count. 3) Finally, thermodynamic and kinetic models coarse-grain to different degrees the molecular mechanisms through which RNAP enters the transcriptional event."

> The problems with this thermodynamic distinction are further revealed by the discussion of model 3 in Figure 1C on page 10. It is claimed that this model shows "true non-equilibrium state-fluxes through promoter states". This assertion is problematic. The only reason the promoter states are away from equilibrium is because the transition between bound RNAP and paused RNAP is treated as irreversible. Irreversible transitions are always away from equilibrium but at the expense of an infinite entropy production. Had the transition been made reversible, as all transitions ultimately are, then the promoter states would still form a tree which continues to satisfy detailed balance (citation [45]). This is hardly a "true non- equilibrium". For that to be plausible, there would have to be non-trivial reversible cycles in the regulatory part of the graph of the Markov process, which would thereby permit finite entropy production during regulation.

This is an excellent point and we thank the reviewer for pointing it out. We completely agree with the reviewer that the notion of perfectly irreversible processes is an idealization that should be wielded carefully, since it destroys potentially important information like entropy production rates. But, to the best of our knowledge using the irreversible idealization is safe for our purposes since we are only calculating state occupation probabilities and not derived quantities like entropy production. Repeating the calculation of fold-change and Fano factor with reversible edges adds algebraic clutter without qualitatively changing the conclusion.

If we did allow reversible edges, in theory the model could reach equilibrium. But putting plausible rate constants on all the edges would, we believe, force the system to a non-equilibrium steady state, since ultimately the various steps that RNAP proceeds through are driven by chemical potentials (ATP vs. ADP, etc.) which are held away from equilibrium. Effectively then, some of the edges of the graph are approximately irreversible. So, we chose the simpler path as often taken in the literature and treated approximate irreversibility as exact.

Ultimately, by calling the state-fluxes "true non-equilibrium," all we meant to convey is that in model 3, detailed balance is guaranteed to be violated *somewhere* in the model. This is rather trivial since there are irreversible edges. That detailed balance is preserved on the repressor edge of the graph is, in light of the separation between "regulation" and "expression" the reviewer explains above, perhaps not surprising.

To address this excellent point we added the following text to the discussion:

“ It is important to mention the potential problems present in all models, including model 5 from Figure 2(A)... Another facet of the models used here is that they can feature irreversible processes characterized by only a single rate process with no corresponding return pathway at odds with thermodynamics. Such models are mathematically convenient and serve only as an approximation to the more realistic situation in which the forward rates are much larger than the backward rates. One consequence is that the inferred rates for the transcription burst rate and burst size can only be thought of as effective rate constants. ...”

Comments on King-Altman diagram method (pages 11, 13) and Hill's work (page 13). I was surprised to see these references. They are historically relevant but the authors seem to be unaware of the modern treatment based on the Matrix-Tree theorem (MTT) in the graph-based approach (citation [44]; for more discussion of Hill's contributions see Biddle et al PRE 101:062125 2020). Theorems of this kind go back to Kirchhoff but the version relevant here was first proved by Bill Tutte in 1948 and is a fundamental result in graph theory. King-Altman and Hill separately and independently found their own versions of this result, couched in ad-hoc languages (“diagrams”). These parochial formulations are divorced from their correct setting within mathematics, making it impossible to see the connections which link mathematics, physics and biology. The authors would be doing a service to the community by referring to the modern formulation and encouraging readers not to get stuck in the past.
The authors make the point that these graph-theoretic methods may explain their calculations, especially the emergence of a “master curve” and the interpretation of its parameters (page 13). I agree with this assertion but the authors may wish to clarify the challenges involved. Specifically, the MTT applies, at present, only to finite graphs. In the context of the present paper, this means the models in Figure 1B, in which RNAP is not explicitly represented and mRNA number is not part of the state. The models in Figure 1C, in which mRNA number is represented within the state, require infinite graphs, for which an extension of the MTT remains an open problem (PMID 32375018), although this may not be such a limitation for determining the mean expression level.

We thank the reviewer for this suggestion. As the paper has been restructured to a shorter format, the original comments involving the King-Altman diagram method were removed. Nevertheless, we think this is a great point to be added in the discussion. We have therefore added the following text:

“ The fact that thermodynamic and kinetic models for the simple repression motif can not only be reconciled, but are indistinguishable from each other at the level of mean mRNA count is a consequence of the possible transitions between promoter microstates. The promoter microstates in all models shown in Figure 1 can explicitly be separated into two groups: 1) promoters with repressor bound, and 2) all other promoters states. The single path between these two groups of states guarantees detailed-balance between the groups. We can therefore separate *expression* related transitions, which by definition must be out of equilibrium, with *regulation* transitions that can in principle be in equilibrium. This implies that the probability distribution of the promoter microstates not involving the repressor take

the same functional form, allowing us to write the fold-change as an effective free energy involving a regulation term, ΔF_R, and a promoter details term, ρ. We offer this argument as a conjecture, and we suspect that a careful argument using the graph-theoretic framework based on the Matrix-Tree Theorem [40], might furnish a "proof" not with some challenges as this theoretical framework applies, at the present, to finite graphs."

> Using the graph-theoretic "linear framework" , as alluded to on page 13. Since I have made this point previously to the senior author, I will not flog a dead horse here but simply draw attention to the several instances above, in which the graph-theoretic Markov process approach helps to clarify what the authors are trying to accomplish.

We thank the reviewer for this suggestion and apologize for being slowing on the uptake in really embracing these methods. Part of the reason we chose PLoS Computational Biology rather than a more specialized physics journal is for its reach to all kind of quantitative-minded biologists that might not necessarily have the necessary background to follow the linear framework. We opted for what we believe is the most transparent and pedagogical way of introducing newcomers to this topic. We hope that the list of references given in the manuscript can serve as a starting point for researchers at all levels of expertise in this field. Further, we really look forward to once and for all engaging with Jeremy to become practitioners of the graph theory approach and to make it a key part of our pedagogy and research in the near future.

4 PLoS Comp. Bio. 04

The authors have investigated the steady-state properties of several elementary gene expression models. A Bayesian framework was used to challenge the models with experimental data. The models were assessed according to (1) their likelihood given the data and (2) the posterior distribution of parameter values. They identified models involving transcriptional bursts as ones that explain the data without introducing poorly determined parameters. They applied the framework to show that transcription factor association and dissociation rates obtained using single-cell expression data are consistent with equilibrium constants obtained using mean population-level data.
Content:
The manuscript is organized into the following sections: 1. Introduction; 2. Mean Gene Expression; 3. Beyond Means in Gene Expression; 4. Finding the "right" model: Bayesian parameter inference; 5. Discussion and future work. There is an additional section 6. Methods, which consists of a link to a github repository. Sections 2 and 3 explore the behaviors of the models and to provide some justification for why a bursty transcription model might be suitable for the present study. Section 4 presents the results of assessing the models and identifying parameter values.

To review section 2, it is helpful to refer to the authors' earlier work in Refs. 32 and 35. In Ref. 32, flow cytometry measurements of single-cell expression of a YFP reporter were obtained from 18 E. coli strains. In each strain, YFP expression was controlled by one of three lac operators (O1, O2, or O3) and one of six different LacI copy numbers. In Ref. 35, the data from Ref. 32 were considered and additional measurements were obtained from lacI point mutants. In these studies, an equation for the fold-change in expression was derived as a function of inducer concentration, repressor copy number, LacI-operator sequence binding energy, the energy difference between the active and inactive states of LacI, and the inducer dissociation constants in the active and inactive states of LacI. The model satisfactorily explained all of the data, as illustrated by collapsing all of the measurements onto a sigmoidal curve where the model is used to compute a new x-axis from the original inputs.

Section 2 essentially asks whether the results of Refs. 32 and 35 would still hold if the model of the promoter were to change. Five models were considered, in which the details of the promoter differ but in which repressor binding still only occurs through a single state. All of these models yield a similar form for the fold-change in expression, with the caveat that the x-axis needs to be shifted by a constant (that is, constant with respect to the variations introduced).

To review sections 3 and 4, it is helpful to refer additionally to the authors' earlier work in Ref. 36 in which single-molecule mRNA FISH measurements of single-cell expression from a number of synthetic promoters were obtained. Constitutive promoter expression was modeled using a Poisson process, and the super-Poisson noise in expression from 18 constitutive promoters, as characterized using the Fano factor ¿ 1, was attributed to quantization error, RNAP copy number variations, and gene copy number variations. Two promoters were placed under control of LacI, which in turn was expressed from an inducible promoter. The dependence of the Fano factor on LacI was measured and the elevation compared to the constitutive expression was explained using a simple model of repression.

Section 3 essentially asks whether the super-Poisson behavior (Fano factor ¿ 1) of the constitutive promoters in Ref. 36 can be explained using an alternative promoter model instead of quantization error, RNAP copy number variations, and gene copy number variations. Of the five models considered, the two bursting models – one coarse-grained and one finer-grained – are the only ones that yield a Fano factor ¿ 1. Here the authors jump ahead a bit, expressing a preference for the coarse-grained model – model 5 – based on Bayesian analysis not yet described. The finer-grained model – model 4 – was ruled out as having parameters that could not be identified.

Finally, section 4 applies the Bayesian framework to analyze the constitutive and repressor-regulated promoter models using data from Ref. 36. The authors immediately rule out the first three constitutive promoter models as having Fano factor ≤ 1. They cite unpublished Ref. 42 to rule out model 4, the finer-grained model, as having parameters that could not be identified using the data. They begin with model 1 as an example to gain intuition, and then move on to analyze model 5, and identify model parameters for 18 constitutive promoters. They find that, given some reasonable assumptions, the repressor binding parameters are consistent with prior equilibrium parameters obtained in refs. 32 and 33, and kinetic rates obtained in ref. 56.

These findings will be of interest to anyone who is interested in quantitative modeling of gene regulation; however issues with the presentation must be addressed before publication as a research article.

Here I insert a quote from the paper:
In reflecting on these 5 models, the reader may feel that exploring a multitude of potential models just to return to a very minimal phenomenological model of bursty transcription may seem highly pedantic.

The authors have hit on an important point here – it is possible to present sections 2-3 much more directly and concisely. In addition, and much more importantly, 2/3 the way into the paper, the reader still does not have the context necessary to identify the main results of the work and to understand their significance. Readers who are not familiar with the authors' previous papers will be lost, and others will be perplexed about how this work connects.

The presentation would be improved substantially by eliminating most of sections 2-3 and refocusing and extending the introduction to motivate the present study in the context of the directly relevant literature rather than in the search for a model that unifies everything since Jacob and Monod. In setting the reader's expectations for the latter, this paper will draw criticism for the limited possibilities explored, and for a narrow focus on the literature. For example, the paper does not really consider the possibilities of bimodal distributions or challenging the dynamical models with time-resolved experimental data. Above I sketched some ideas for how to draw a thread through this study, and the authors might have different ideas. The main thing is to help readers to understand precisely how the present work builds on and extends previous work. The discussion would be the place for the authors to speculate on the implications of their work for achieving larger goals, after the reader knows what they have done.

We thank the reviewer for this excellent suggestion. Indeed, all reviewers came to the same conclusion that the way that the work was presented could be shortened enormously, clarifying at the same time the novel contributions of this publication. We have substantially altered the structure of the paper based on this thoughtful comment, shortening sections 2 and 3. We tried really hard to convey the logical flow between the sections, hopefully making it easier for readers not familiar with our previous work to follow the manuscript.

For example, in section 2, the essential result is that the coarse-grained bursty model (model 5 in Fig. 1C) yields the same fold-change expression as the elementary promoter model (model 1 in Fig. 1C), so that switching to this model doesn't change the meaning of parameters obtained, e.g., in refs. 32 and 35. This point could be made without any math, simply by noting that both models 1 and 5 are equivalent from the perspective of repressor binding (both have only a single repressor-free state). Models 2, 3, and 4 don't need to be considered at all, and the same goes for the models in Fig. 1B; insofar as the authors need to refer to them in this paper, they can cite the literature.

We appreciate this suggestion by the reviewer. As a result we have rewritten and substantially reduced section 2, listing the main results, and directing interested readers to the

supplementary material. As written now, we think it captures the essence of what the reviewer is proposing here, though we prefer to keep the different models since this entire suite of models have been part of the conversation in the field and we think it is helpful to collect the results of each all in one place.

> Similarly, in section 3, only models 1 and 5 need to be considered. Whereas the authors' prior work in Ref. 36 using model 1 attributed the super-Poisson noise to extrinsic effects, here they bring the super-Poisson behavior into the model itself. The authors really only need to cite prior work describing how transcriptional bursting leads to a Fano factor ¿ 1, and to note that whereas elsewhere the bursting model is applied to describe regulated expression (e.g. Munsky et al 2012, `https://science.sciencemag.org/content/336/6078/183.full`), here it is used to describe constitutive expression from the promoters in Ref. 36 (citing other work where a similar approach was taken, if there is any).
>
> I offer the above ideas merely as suggestions of an approach to address the presentation issues. The authors might come up with a different approach; however, I must stress here that that the only way I could make sense of what was going on in this paper was to go back to the literature for the context, to ignore much of the exposition in the manuscript (which reads much more like a monograph from an intelligent and curious mind than a research paper), and to focus on the figures and the corresponding explanatory text. Fixing this problem will be essential if the important parts of this paper are to reach the intended audience.

The ideas presented are well taken. We are grateful to the reviewer for suggesting this restructuring of the manuscript. We are very happy with the current state, adapting all suggestions from the reviewers in order to make the punchline of the manuscript much more clear, while still keeping the level of pedagogy that we were aiming for originally.

> Some details in Section 4 are missing. For example, there are no results to back up the conclusion that the parameters in model 4 cannot be identified. In addition, the Oid, O1, and O2 subscripts are introduced without information about what they correspond to; they are described in Figure 4 as operators, but some readers still won't know what this means. This key section should be revised with an eye towards providing the reader with all of the evidence required to back up the conclusions, and defining all of the elements of the repression model.

We thank the reviewer for bringing up these concerns. We apologize that the message didn't come across as clearly as it should have. The reason why this model is not considered is because the rate parameters cannot be uniquely identified. In a recent publication, we applied a similar Bayesian inference pipeline to the one presented here and found a degeneracy in the parameters. Specifically in Figure S2 of [8] (reproduced here as Figure R4), a so-called corner plot intended to show 2D slices of the joint probability distribution between parameters, shows that the promoter off rate k^p_{off} (k^- in this manuscript) and the mRNA production

rate r_m (r in this manuscript) cannot be uniquely identified.

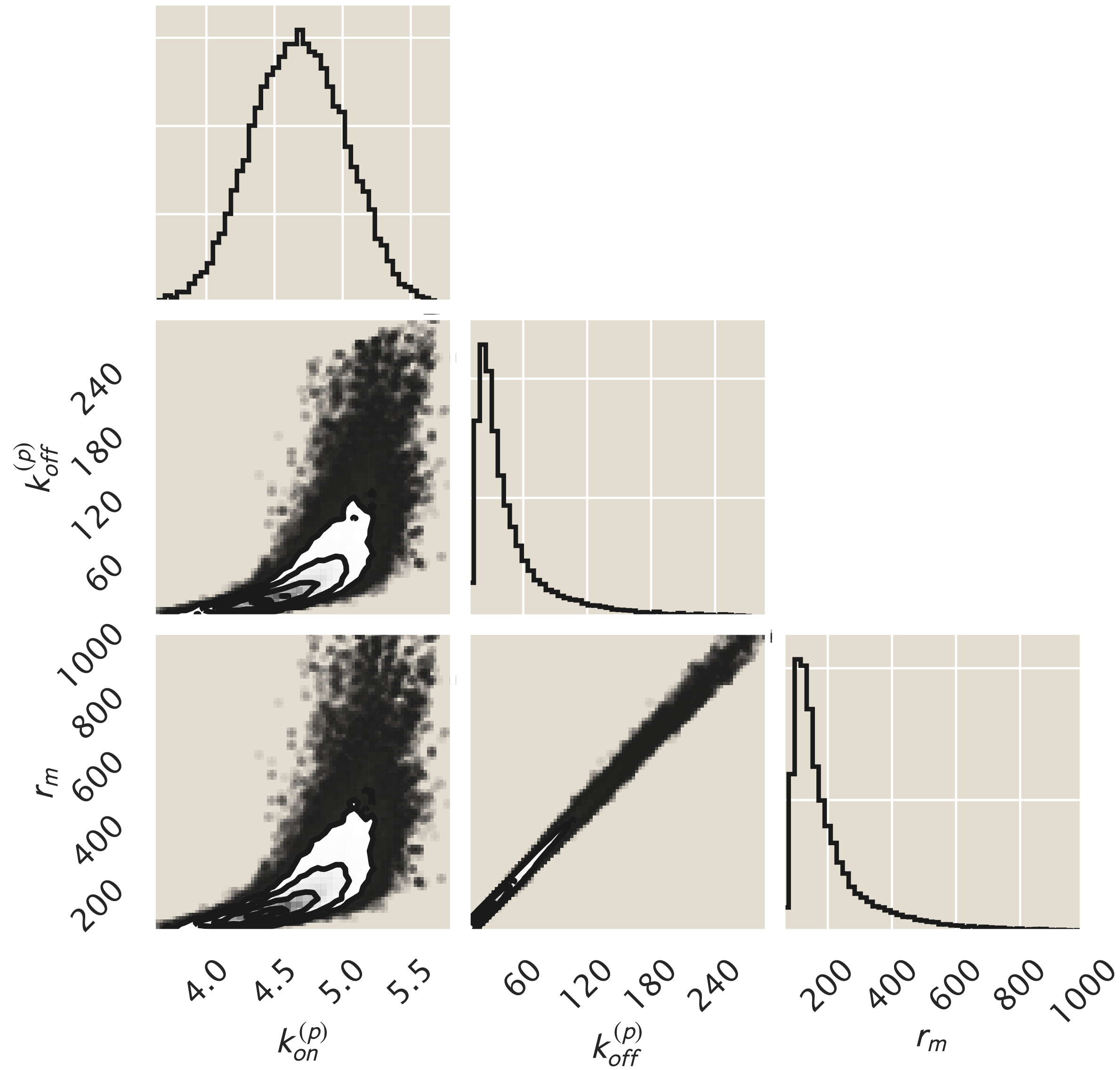

Figure R4. MCMC posterior distribution for model 5. 2D and 1D projections of the 3D parameter space. The parameter values are (in units of the mRNA degradation rate γ_m) $k_{on}^{(p)} = 4.3^{+1}_{-0.3}$, $k_{off}^{(p)} = 18.8^{+120}_{-10}$ and $r_m = 103.8^{+423}_{-37}$ which are the modes of their respective distributions, where the superscripts and subscripts represent the upper and lower bounds of the 95th percentile of the parameter value distributions.

This issue with the model is mentioned in the main text in the following sections: In Section 3, while discussing the Fano factor for different models, we mention the drawbacks from model 4, citing a recent publication where we explored this issue in depth [8]:

"While this seems promising, there is a major drawback for our purposes which was already uncovered by the authors of [8]: the statistical inference problem is nonidentifiable, in the

sense described in Section 4.3 of [41]. What this means is that it is impossible to infer the parameters r and k^- from the single-cell mRNA counts data of [11] (as shown in Fig. S2 of [8]).”

Later in that section, when we introduce the advantages of model 5, we again point at model 4 “sloppy” nature where only combinations of parameters can be uniquely identified:

“The final model we consider is inspired by the failure mode of model 4. The key observation above was that, as found in [8], only two parameters, k^+ and the ratio r/k^-, could be directly inferred from the single-cell mRNA data from [11].”

In Section 4 we again point the reader to our previous analysis of model 4 when we say:

“We will also not explicitly consider model 4 from Figure 2(A) since it was already thoroughly analyzed in [8], and since model 5 can be viewed as a special case of it.”

We also thank the reviewer for highlighting the fact that we didn’t define what an operator is. We have now added the following colored text to Section 4.

“Before showing the mathematical formulation of our statistical inference model, we would like to sketch the intuitive structure. The dataset from [11] we consider consists of single-cell mRNA counts data of nine different conditions, spanning several combinations of three unique repressor binding sites (the so-called Oid, $O1$, and $O2$ operators) and four unique repressor copy numbers.”

> In addition, the paper currently intermixes methods, results, and discussion in sections 2-4, with additional discussion in section 5. The methods should be broken out into a separate section, to follow the journal guidelines (there is no methods content in section 6 right now). The clarity of the exposition of the results would be improved by moving most of the interpretive comments and caveats to the discussion section or the supplementary material, as appropriate.

We agree that the intermixing of the discussion in between sections could have confused a lot of potential readers. We have now sent all of the relevant points to be discussed to the end of the manuscript. Again, we are enormously thankful to the reviewer for the excellent tips on how to restructure the manuscript.

> Some specific comments on the current manuscript follow. Some of these might refer to material that would be better left out of a revision, as described above.
>
> p. 3, para 2. It would be good to walk the reader through the other panels of Fig. 1 in order here. As written Fig. 1B remains unaddressed, and Figs. 1C and 1D are mentioned but not explained.

We thank the reviewer for highlighting this inconsistency. In section 2 we now have the following paragraph, addressing this issue:

"'Figure 1(B) and (C) depicts a smorgasbord of mathematicized cartoons for simple repression using both thermodynamic and kinetic models, respectively, that have appeared in previous literature. For each cartoon, we calculate the fold-change in mean gene expression as predicted by that model, deferring most algebraic details to Appendix 1. What we will find is that for all cartoons the fold-change can be written as a Fermi function of the form

$$\text{fold-change} = \left(1 + \exp(-\Delta F_R + \log(\rho))\right)^{-1}, \tag{1}$$

where the effective free energy contains two terms: the parameters ΔF_R, an effective free energy parametrizing the repressor-DNA interaction, and ρ, a term derived from the level of coarse-graining used to model all repressor-free states. In other words, the effective free energy of the Fermi function can be written as the additive effect of the regulation given by the repressor via ΔF_R, and the kinetic scheme used to describe the steps that lead to a transcriptional event via $\log(\rho)$ (See Figure 1(D), left panel). This implies all models collapse to a single master curve as shown in Figure 1(D). We will offer some intuition for why this master curve exists and discuss why at the level of the mean expression, we are unable to discriminate "right" from "wrong" cartoons given only measurements of fold-changes in expression."'

> p. 3, para 2. "...the same underlying functional form..." ΔF is undefined in the equation. Please define in the Fig. 1 caption or here.

We thank the reviewer for this great suggestion. This paragraph in the introduction now reads as

"The logic of the remainder of the paper is as follows. In section 2, we show how both thermodynamic models (Figure 1(B)) and kinetic models based upon the chemical master equation (Figure 1(C)) all culminate in the same underlying functional form for the fold-change in the average level of gene expression with an effective free energy ΔF_R capturing the regulation given by the transcription factor, and a term ρ describing the level of coarse-graining of the transcriptional events as shown in Figure 1(D)."

p. 3, Section 2. The main result of this section is that all of the models considered yield a similar form for the fold-change in expression, with the x-axis is shifted by a constant $\log \rho$ (that is, constant with respect to the variations introduced). Instead of walking through all of the derivations, it is possible to argue logically instead, observing the structure of the models in Fig. 1. The repressor-bound state (call it state 1) can only be reached from a single repressor-free state (call it state 0). The differences in the models are associated with the additional states of the repressor-free promoter, which draw density from states 0 and 1. Because the population ratio of states 0:1 must be the same in all of the models, the population ratio of (all repressor-free states):1 is greater than or equal to the 0:1 ratio. The difference in the log ratio is equal to $\log \rho$. Model 5, like model 1, only consists of a single repressor-free state, which is why $\rho = 1$ for both of these models; values of the equilibrium constant obtained using models 1 and 5 therefore should be comparable. The authors might be able to find something to cite about this type of argument in a textbook or elsewhere in the literature.

We thank the reviewer for this incredibly intuitive argument. We agree that this is an excellent way to generalize the findings of Section 2. For this resubmission we shortened the section substantially, and included this and another reviewer's equally great intuition into our explanation as:

"Again, we consign all details of the derivation to Appendix 1. Here we just highlight the general findings for all five kinetic models. As already shown in Figure 1(C) and (D), all the kinetic models explored can be collapsed onto the master curve. Given that the repressor-bound state only connects to the rest of the promoter dynamics via its binding and unbinding rates, k_R^+ and k_R^- respectively, all models can effectively be separated into two categories: a single repressor-bound state, and all other promoter states with different levels of coarse graining. This structure then guarantees that, at steady-state, detailed balance between these two groups is satisfied. What this implies is that the steady-state distribution of each of the non-repressor states has the same functional form with or without the repressor, allowing us to write the fold-change as a product of the ratio of the binding and unbinding rates of the promoter, and the promoter details. This is

$$\text{fold-change} = \left(1 + \frac{k_R^+}{k_R^-}\rho\right)^{-1}, \tag{2}$$

$$= \left(1 + \exp(-\Delta F_R + \log(\rho))\right)^{-1}, \tag{3}$$

where $\Delta F_R \equiv -\log(k_R^+/k_R^-)$, and the functional forms of ρ for each model change as shown in Figure 1(C). Another intuitive way to think about these two terms is as follows: in all kinetic models shown in Figure 1(C) the repressor-bound state can only be reached from a single repressor-free state. The ratio of these two states –repressor-bound and adjacent repressor-free state– must remain the same for all models, regardless of the details included in other promoter states if ΔF_R represents an effective free energy of the repressor binding the DNA operator. The presence of other states then draws probability density from the promoter being in either of these two states, making the ratio between the repressor-bound state and *all* repressor-free states different. The log difference in this ratio is given by $\log(\rho)$.

Since model 1 and model 5 of Figure 1(C) consist of a single repressor-free state, ρ is then necessarily 1 (See Appendix 1 for further details)."

> p. 3, below Eq. 1. "...the models differ along several key axes: what states to consider and how to compute the probabilities of those states" In what sense are these axes? Or do the authors instead mean that the models differ in key aspects/characteristics.

We apologize for this confusion. We definitely meant axes in the spirit implied by the reviewer. We have now changed axes to aspects.

> p. 3, para 4. "As seen in Figure 1(B), even within the class of thermodynamic models..." Either use a consistent terminology equilibrium/thermodynamic or explain somewhere that they are being used to describe the same thing.

We thank the reviewer for demanding consistency in the language we use. We agree that the way we utilize the terminology of equilibrium/thermodynamic and non-equilibrium/kinetic was inconsistent. Furthermore, thanks to reviewer 2 we decided that the use of non-equilibrium in this context can be inappropriate from a physics perspective. We have therefore changed all uses of equilibrium to thermodynamic and non-equilibrium to kinetic –even in the title of the manuscript.

> p. 5, para 2. "The second equality in Eq. 2 follows from assuming that the translation efficiency, i.e., the number of proteins translated per mRNA, is the same in both conditions." It looks like there is no assumption about translation in the equation. Where does the assumption enter?

We thank the reviewer for highlighting this point. We mentioned this because in previous publications [13], [18] we measured gene expression using genetically encoded fluorescent reporters, measured at the level of protein. But it is true that in the equation we showed the ratio of mRNA copy numbers. We have therefore modified the equation to be

$$\text{fold-change} = \frac{\langle \text{gene expression with repressor present} \rangle}{\langle \text{gene expression with repressor absent} \rangle} = \frac{\langle m(R > 0) \rangle}{\langle m(R = 0) \rangle} = \frac{\langle p(R > 0) \rangle}{\langle p(R = 0) \rangle}, \quad (4)$$

adding the proper definition of the variables in the main text.

> p. 5, last para. "...ρ which subsumes all details of transcription in the absence of repressors." The meaning of ρ isn't so clear yet. Can the authors try to explain it to the reader earlier, beyond the fact that it subsumes the details of transcription? Also aren't the authors assuming that ρ captures the details of transcription both in the presence and absence of repressor?

We agree with the reviewer premise that the readers will benefit from an earlier exposition of what the term ρ captures. The previously mentioned paragraph from Section 2 where we modified the text to describe Figures 1C and 1D contains a longer explanation we reproduce here to highlight it:

"Figure 1(B) and (C) depicts a smorgasbord of mathematicized cartoons for simple repression using both thermodynamic and kinetic models, respectively, that have appeared in previous literature. For each cartoon, we calculate the fold-change in mean gene expression as predicted by that model, deferring most algebraic details to Appendix 1. What we will find is that for all cartoons the fold-change can be written as a Fermi function of the form

$$\text{fold-change} = (1 + \exp(-\Delta F_R + \log(\rho)))^{-1}, \quad (5)$$

where the effective free energy contains two terms: the parameters ΔF_R, an effective free energy parametrizing the repressor-DNA interaction, and ρ, a term derived from the level of coarse-graining used to model all repressor-free states. In other words, the effective free energy of the Fermi function can be written as the additive effect of the regulation given by the repressor via ΔF_R, and the kinetic scheme used to describe the steps that lead to a transcriptional event via $\log(\rho)$ (See Figure 1(D), left panel). This implies that all models collapse to a single master curve as shown in Figure 1(D). We will offer some intuition for why this master curve exists and discuss why at the level of the mean expression, we are unable to discriminate "right" from "wrong" cartoons given only measurements of fold-changes in expression."

> p. 6, Eq. (3). The variables in this equation should be described immediately afterwards.

We apologize for this omission in the original submission. As the paper stands now, this section was removed as part of the shortening of the document. For this resubmission we made sure that all variables are immediately defined after the equations.

> p. 9, para 3. "In fact, we suspect any model in which transcription proceeds through a multistep cycle must necessarily have $\nu < 1$." See, e.g., Floyd et al (2010) Biophys J 99:360 (`https://www.ncbi.nlm.nih.gov/pmc/articles/PMC2905077/`) and refs therein.

We thank the reviewer for pointing out this very interesting reference. The pedagogical style in which it is written made it a very fun read. The point brought up in the paper has to do with the study of dwell-times. The relevant distribution we would have to observe to apply this theoretical framework is the time between transcription events. From that we could potentially use this framework to learn something about the number of kinetic steps involved in transcription initiation. But this would require live imaging with something like an MS2 construct [42] rather than single-molecule FISH counts. Furthermore, as we understand it, this framework does not address the noise in the downstream product, i.e., the noise in mRNA counts. What this framework is concerned about is the variability in the

time between events, not the variability in the number of events themselves. Nevertheless the idea is intriguing, and we will definitely keep in mind this approach for further refinements of our model.

> p. 21, para 2. See also Senecal et al, 2104 (`https://www.sciencedirect.com/science/article/pii/S2211124714004471?via\%3Dihub`, doi:10.1016/j.celrep.2014.05.053) and Friedman et al, 2006 (`https://doi.org/10.1103/PhysRevLett.97.168302`).

We thank the reviewer again for these interesting suggestions. We have added the paper by Senecal to our list of references when discussing gene expression bursting. It is very interesting to us that we find their approach philosophically very different to what we are trying to do in this publication. The idea presented here is that we look for the level of coarse-graining that satisfies our physical intuition of how the gene regulation is taking place. This is the reason we limited this study to the exploration of the simple repression motif. We wanted a system in which we have complete knowledge of the regulatory architecture and the relevant molecular players involved in here. While Senecal takes an approach in which a two-state model (equivalent to model 4 in Figure 2(A)) guides their intuition for the role that transcription factors playing in the regulation. We are thankful to the reviewer for showing us this alternative way of tackling the problem.

> p. 26, Eq. (52). See Paulsson and Ehrenberg, 2000 (`https://journals.aps.org/prl/pdf/10.1103/PhysRevLett.84.5447`). Also see Friedman et al, 2006 (`https://doi.org/10.1103/PhysRevLett.97.168302`) which derives a continuous limit.

Unfortunately, we were not clear on how to implement the insights from these references in the context of our paper. We hope the reviewer understands that we are not trying to ignore him, but rather simply were unclear on what to do.

> The data for the publication have been uploaded to github at `https://github.com/RPGroup-PBoC/bursty_transcription`. As it stands it's not clear how to reproduce the results in the paper using what's provided. There is code in the repo but the documentation could be improved, e.g., to indicate which scripts do what, and to indicate external dependencies.

We are grateful that the reviewer thoroughly checked all the material we made available. We have now corrected the structure of the GitHub repository, annotating the directories and their content in order to make the work much more reproducible. In particular the `README` file in the `code` directory lists all of the Python dependencies used for the analysis.

References

[1] J. Q. Boedicker, H. G. Garcia, and R. Phillips, "Theoretical and Experimental Dissection of DNA Loop-Mediated Repression," *Phys. Rev. Lett.*, vol. 110, no. 1, p. 018 101, 2013.

[2] Y.-J. Chen, S. Johnson, P. Mulligan, A. J. Spakowitz, and R. Phillips, "Modulation of DNA loop lifetimes by the free energy of loop formation," *PNAS*, vol. 111, no. 49, pp. 17 396–17 401, 2014.

[3] J. E. Keymer, R. G. Endres, M. Skoge, Y. Meir, and N. S. Wingreen, "Chemosensing in Escherichia coli: two regimes of two-state receptors.," *PNAS*, vol. 103, no. 6, pp. 1786–91, 2006.

[4] B. M. Martins and P. S. Swain, "Trade-Offs and Constraints in Allosteric Sensing," *PLoS Comput. Biol.*, vol. 7, no. 11, e1002261, 2011.

[5] T. Einav, L. Mazutis, and R. Phillips, "Statistical Mechanics of Allosteric Enzymes," *J. Phys. Chem. B*, vol. 120, no. 26, pp. 6021–6037, 2016.

[6] S. Chong, C. Chen, H. Ge, and X. S. Xie, "Mechanism of transcriptional bursting in bacteria," *Cell*, vol. 158, no. 2, pp. 314–326, Jul. 2014.

[7] S. A. Sevier, D. A. Kessler, and H. Levine, "Mechanical bounds to transcriptional noise," *Proceedings of The National Academy Of Sciences Of The United States Of America*, vol. 113, no. 49, pp. 13 983–13 988, Dec. 2016.

[8] M. Razo-Mejia, S. Marzen, G. Chure, R. Taubman, M. Morrison, and R. Phillips, "First-principles prediction of the information processing capacity of a simple genetic circuit," *Phys. Rev. E*, vol. 102, 2020.

[9] S. Proshkin, A. R. Rahmouni, A. Mironov, and E. Nudler, "Cooperation Between Translating Ribosomes and RNA Polymerase in Transcription Elongation," *Science (80-.).*, vol. 328, no. 5977, pp. 504–508, 2010.

[10] H. Chen, K. Shiroguchi, H. Ge, and X. S. Xie, "Genome-wide study of mRNA degradation and transcript elongation in *Escherichia coli*," *Molecular Systems Biology*, vol. 11, no. 1, p. 781, Jan. 2015.

[11] D. L. Jones, R. C. Brewster, and R. Phillips, "Promoter architecture dictates cell-to-cell variability in gene expression," *Science*, vol. 346, no. 6216, pp. 1533–1536, Dec. 2014.

[12] L.-h. So, A. Ghosh, C. Zong, L. A. Sepúlveda, R. Segev, and I. Golding, "General properties of transcriptional time series in *Escherichia coli*," *Nature Genetics*, vol. 43, no. 6, pp. 554–560, Jun. 2011.

[13] M. Razo-Mejia, S. L. Barnes, N. M. Belliveau, G. Chure, T. Einav, M. Lewis, and R. Phillips, "Tuning transcriptional regulation through signaling: A predictive theory of allosteric induction," *Cell Systems*, vol. 6, no. 4, 456–469.e10, Apr. 2018.

[14] G. Chure, M. Razo-Mejia, N. M. Belliveau, T. Einav, Z. A. Kaczmarek, S. L. Barnes, M. Lewis, and R. Phillips, "Predictive shifts in free energy couple mutations to their phenotypic consequences," *Proceedings of The National Academy Of Sciences Of The United States Of America*, vol. 116, no. 37, pp. 18 275–18 284, Sep. 2019.

[15] A. Senecal, B. Munsky, F. Proux, N. Ly, F. E. Braye, C. Zimmer, F. Mueller, and X. Darzacq, "Transcription Factors Modulate c-Fos Transcriptional Bursts," *Cell Rep.*, vol. 8, no. 1, pp. 75–83, 2014.

[16] L. Weber, W. Raymond, and B. Munsky, "Identification of gene regulation models from single-cell data," *Phys. Biol.*, vol. 15, no. 5, p. 055 001, 2018.

[17] G. Chure, Z. A. Kaczmarek, and R. Phillips, "Physiological adaptability and parametric versatility in a simple genetic circuit," *bioRxiv*, pp. 10.1101/2019.12.19.878462, Dec. 2019.

[18] H. G. Garcia and R. Phillips, "Quantitative dissection of the simple repression input-output function," *Proceedings of The National Academy Of Sciences Of The United States Of America*, vol. 108, no. 29, pp. 12 173–12 178, Jul. 2011.

[19] L. Bintu, N. E. Buchler, H. G. Garcia, U. Gerland, T. Hwa, J. Kondev, and R. Phillips, "Transcriptional regulation by the numbers: Models," *Current Opinion in Genetics & Development*, vol. 15, no. 2, pp. 116–24, 2005.

[20] H. G. Garcia, J. Kondev, N. Orme, J. A. Theriot, and R. Phillips, "Thermodynamics of Biological Processes," in *Methods in Enzymology*, vol. 492, Elsevier, 2011, pp. 27–59.

[21] R. Phillips, "Napoleon is in equilibrium," *Annual Review of Condensed Matter Physics*, vol. 6, no. 1, pp. 85–111, Mar. 2015.

[22] R. Phillips, N. M. Belliveau, G. Chure, H. G. Garcia, M. Razo-Mejia, and C. Scholes, "Figure 1 theory meets figure 2 experiments in the study of gene expression," *Annual Review of Biophysics*, vol. 48, no. 1, pp. 121–163, May 2019.

[23] G. K. Ackers, A. D. Johnson, and M. A. Shea, "Quantitative model for gene regulation by lambda phage repressor," *Proceedings of The National Academy Of Sciences Of The United States Of America*, vol. 79, no. 4, pp. 1129–33, 1982.

[24] M. A. Shea and G. K. Ackers, "The OR control system of bacteriophage lambda. A physical-chemical model for gene regulation," *Journal of Molecular Biology*, vol. 181, no. 2, pp. 211–30, 1985.

[25] N. E. Buchler, U. Gerland, and T. Hwa, "On schemes of combinatorial transcription logic.," *Proceedings of The National Academy Of Sciences Of The United States Of America*, vol. 100, no. 9, pp. 5136–5141, Apr. 2003.

[26] J. M. Vilar and S. Leibler, "DNA looping and physical constraints on transcription regulation," *Journal of Molecular Biology*, vol. 331, no. 5, pp. 981–9, 2003.

[27] J. M. Vilar, C. C. Guet, and S. Leibler, "Modeling network dynamics: The *lac* operon, a case study," *Journal of Cell Biology*, vol. 161, no. 3, pp. 471–6, 2003.

[28] L. Bintu, N. E. Buchler, H. G. Garcia, U. Gerland, T. Hwa, J. Kondev, T. Kuhlman, and R. Phillips, "Transcriptional regulation by the numbers: Applications," *Current Opinion in Genetics & Development*, vol. 15, no. 2, pp. 125–135, Apr. 2005.

[29] J. Gertz, E. D. Siggia, and B. A. Cohen, "Analysis of combinatorial *cis*-regulation in synthetic and genomic promoters," *Nature*, vol. 457, no. 7226, pp. 215–8, 2009.

[30] M. S. Sherman and B. A. Cohen, "Thermodynamic state ensemble models of *cis*-regulation," *PLoS Computational Biology*, vol. 8, no. 3, e1002407, 2012.

[31] J. M. Vilar and L. Saiz, "Reliable prediction of complex phenotypes from a modular design in free energy space: An extensive exploration of the *lac* operon," *ACS Synthetic Biology*, vol. 2, no. 10, pp. 576–86, 2013.

[32] M. S. Ko, "A stochastic model for gene induction," *Journal of Theoretical Biology*, vol. 153, no. 2, pp. 181–94, 1991.

[33] J. Peccoud and B. Ycart, "Markovian modelig of gene product synthesis," *Theoretical Population Biology*, vol. 48, pp. 222–234, 1995.

[34] M. T. J. Record, W. Reznikoff, M. Craig, K. McQuade, and P. Schlax, "*Escherichia coli* RNA polymerase (sigma70) promoters and the kinetics of the steps of transcription initiation," in *In Escherichia coli and Salmonella Cellular and Molecular Biology*, F. C. Neidhardt, R. C. III, J. L. INGRAHAM, *et al.*, Eds., Washington, DC: ASM Press, 1996, pp. 792–821.

[35] T. B. Kepler and T. C. Elston, "Stochasticity in transcriptional regulation: Origins, consequences, and mathematical representations.," *Biophysical Journal*, vol. 81, pp. 3116–36. 2001.

[36] A. Sanchez and J. Kondev, "Transcriptional control of noise in gene expression," *Proceedings of The National Academy Of Sciences Of The United States Of America*, vol. 105, no. 13, pp. 5081–6, 2008.

[37] V. Shahrezaei and P. S. Swain, "Analytical distributions for stochastic gene expression," *Proceedings of The National Academy Of Sciences Of The United States Of America*, vol. 105, no. 45, pp. 17 256–17 261, Nov. 2008.

[38] A. Sanchez, H. G. Garcia, D. Jones, R. Phillips, and J. Kondev, "Effect of promoter architecture on the cell-to-cell variability in gene expression," *PLoS Computational Biology*, vol. 7, no. 3, e1001100, 2011.

[39] D. Michel, "How transcription factors can adjust the gene expression floodgates," *Progress in Biophysics and Molecular Biology*, vol. 102, no. 1, pp. 16–37, 2010.

[40] J. Gunawardena, "A linear framework for time-scale separation in nonlinear biochemical systems," *PLoS ONE*, vol. 7, no. 5, K. Selvarajoo, Ed., e36321, May 2012.

[41] A. Gelman, J. B. Carlin, H. S. Stern, D. B. Dunson, A. Vehtari, and D. B. Rubin, *Bayesian Data Analysis*, Third edition, ser. Chapman & Hall/CRC Texts in Statistical Science. Boca Raton: CRC Press, 2013.

[42] I. Golding, J. Paulsson, S. M. Zawilski, and E. C. Cox, "Real-time kinetics of gene activity in individual bacteria," *Cell*, vol. 123, no. 6, pp. 1025–1036, Dec. 2005.
