# Supplementary material for: Reconciling kinetic and thermodynamic models of bacterial transcription
Source: PLoS Comput Biol. 2021 Jan 19;17(1):e1008572. doi: 10.1371/journal.pcbi.1008572 (PMC7845990; doi:10.1371/journal.pcbi.1008572)
Supplement: Attachment — Submitted filename: plos-cb-phillips-reconciling-kinetic-equilibrium-models-revision-review.pdf [file pcbi.1008572.s005.pdf]

PCOMPBIOL-D-20-01024R1
Reconciling kinetic and equilibrium models of bacterial transcription
Morrison, Razo-Meija, Phillips

General comments

The authors have made a conscientious effort to address the comments of all reviewers, for which I commend them. I appreciate the care and attention to detail which the authors have shown in explaining their responses. I am generally happy with the changes made to address the specific points I raised previously. I also feel that the reorganisation of the paper has significantly improved its clarity and readability. I would make one further comment on the topic of being away from equilibrium. In my view, the authors' change of language from “equilibrium” and “non-equilibrium” to “thermodynamic” and “kinetic”, respectively, while it helps, does not get to the heart of the matter. Perhaps a better way to articulate this is to note that, from the graph-theoretic viewpoint, these two conditions are extremes. Given some choice of a cycle basis for a graph, some cycles may remain at thermodynamic equilibrium (meaning that they continue to satisfy the cycle condition, with no entropy production), while other cycles may not be at equilibrium. Depending on the cycles and how far they are from equilibrium, quite different functional properties can emerge. I think this offers a more inclusive and productive language in which to explore the non-equilibrium landscape. However, I agree that it may be conceptual overkill to adopt this view for the relatively simple models considered here. On that note, I am happy to recommend publication of this revised version.

Jeremy Gunawardena
