# Supplementary material for: Reconciling kinetic and thermodynamic models of bacterial transcription
Source: PLoS Comput Biol. 2021 Jan 19;17(1):e1008572. doi: 10.1371/journal.pcbi.1008572 (PMC7845990; doi:10.1371/journal.pcbi.1008572)
Supplement: Attachment — Submitted filename: Review2_Morrison_PCB.pdf [file pcbi.1008572.s006.pdf]

The authors revised manuscript is much improved from the previous version, and I found it to be more compelling. My biggest concern about the previous manuscript was that it did not provide a clear context of the author's contributions in light of what else had been done in the many fields that were touched on in this long and complicated article. The revised version is better on that account, such that it is now easier to follow the thread of their arguments. In particular, sections (2) and (3) are now much better linked to one another. The citations are more thorough than before, although there are still a couple places in which references could be added to improve the contextualization of the authors' results or to reduce the length of the manuscript (see below). The results are strong and convincing – at least, all except for those in Fig 4(B), which still seems sketchy to my understanding. More detailed comments are below.

Major Comments

(1) In my previous review, I may have been hasty and perhaps unfair in my criticism of the 'non-surprising' nature of their result for the collapse of the mean fold-change function. Upon my original reading, this seemed to be an obvious fact that would stem from (1) the nature of discrete state Markov processes, which allows one to write the steady state of the master equation as the normalized null vector of an infinitesimal generator matrix and (2) the authors' construction that there is a single path between two disjoint sets of bound and unbound repressor states. However, after a short search online, I did not find this exact result in the literature, and although it is straightforward to derive their result for a general discrete state Markov chain using many different approaches, the algebra needed to get it the clean form presented by the authors is not trivial and the insight provided by that form is valuable. Therefore, I will retract my previous statement about 'non-surprising' results and apologize to the authors for the harshness of my previous comments. (As an aside, I fully agree that surprise should not be a criteria of scientific review.)

(2) The authors were correct to call me out on my evident annoyance at their earlier manuscript. Indeed, I was indeed upset by the earlier manuscript's (i) lack of citations to existing literature dealing with the inference of multi-state kinetic models using single-cell variations in gene expression and (ii) excessive length and use of informal phrasing.

Although I stand by my comments that the original manuscript lacked proper citations, I could have raised these objections in a kinder manner (and with more specific instruction rather than sending a massive laundry list of authors for them to consider). In any case, the referencing and writing is now much improved in the new version, but I would still strongly recommend the inclusion of the following two papers as they are relevant to the work shown here:

[Raj, et al, PLoS Biology, 4(10): e309, 2006] examines maximum likely estimation of bursting gene regulation models using single-molecule FISH data and as a

function of inducer concentration. To my knowledge, this is the first paper to do this kind of parameter estimation.

[Kumar N, et al, PLoS Comput Biol 11(10): e1004292, 2015] discusses the geometric burst distribution that arises from different versions of the random telegraph model (section 3.1.4 in current manuscript). They cite earlier papers that use the approximation, and to be honest, I do not know what was the first paper to derive the burst distribution.

(3) The introduction and discussion of Section (2) regarding thermodynamic models could use more motivational text for (a) why one would want to consider thermodynamic models (TM) rather than kinetic models (KM) and (b) how one is limited in choosing thermodynamic models.

As a non-physicist, I can see two important benefits (I am sure the authors can list more): (i) the number of parameters in TM scales only with the number of states (N), whereas KM models scale with the number of interactions ($N^2 - N$), and (ii) TM models can be described without requiring a topology, whereas KM need to know the full reaction topology. These benefits are implied by Fig. 1, but it would help to have them spelled out more explicitly along with other benefits that the authors consider important.

On the other hand, enumerating the benefits of KM would also be beneficial: (i) KM allow for analysis of transient phenomena with temporal changes in conditions whereas TM can only explore quasi-steady state phenomena, and (ii) KM allow for the exploration of probabilistic behavior. The latter of these is mentioned in the text, but the former should be highlighted as well (especially given the plethora of single-cell experiments done in transient environments).

(4) The transition from Section (2) to Section (3) is much better than in the previous version, but could still be better motivated using existing literature for noisy gene expression (thus, the laundry list of authors I provided in my previous review for people who have integrated stochastic analyses with single-cell data for model identification). My biggest concern with the previous manuscript was that the authors presented their discovery to a collapse of the mean out of context with the fact that many other researchers (1) have noted a similar lack of information contained within mean values alone, and (2) have taken a similar approach to including variability as an added phenotype to help improve model identification. Observations in previous literature abound over the past 15 years that show the means are insufficient for identification, whereas including variance or distributions provide much better constraints on models and parameters. Indeed, that was the point of the first half and Fig. 2 (mean and Fano factor versus the k_+ and k_-) of our old 2012 review [Munsky, Science, 2012], which is itself almost identical to Fig. 4 in an earlier bursting paper [Iyer-Biswas, Phys, Rev E, 2009] (I appreciate that both of these papers are now included in the authors citation list). Acknowledging this research trend (i.e., toward incorporation of variability in the identification of kinetic models) in the transition from Section (2) to Section (3) would go a long way to satisfying my concern on this account.

(5) In Section (3), the authors show that various model sets are distinguishable from one another via the fact that some permit Fano Factors greater than one while others do not. Do the authors find any collapse of the models' Fano Factors that does not depend on limiting cases of the parameters? Or, are all models under consideration now resolvable by measuring their Fano factors at different induction levels? If the authors, could flesh out this issue a little more, it would be valuable.

(6) The geometric bursting model in section 3.1.4 was studied nicely in previous work, so this section can safely be reduced or moved to the SI to save space. As a side note, the derivation that the burst size is a geometric distribution can be derived directly by considering a coin toss experiment (heads = transcript is mad and you can toss the coin again; tails = game over). If looking for papers that use this model, please consider the following paper (and several earlier papers referenced within):

Kumar N, et al, Transcriptional Bursting in Gene Expression: Analytical Results for General Stochastic Models, PLoS Comput Biol 11(10): e1004292

(7) Figure 4B is still not at all convincing to me, and to my mind this is now the weakest part of the manuscript. The top panel shows two estimates of a parameter at about 1.1 and 1.2 (differing by 10%) on the x-axis and that are compared to another estimate of the equivalent parameters, which seem to range from 1.4 to 2.8 on the y-axis. In all cases, the uncertainty bars cannot reconcile the difference (i.e., they do not extend to cross the diagonal line). I would have thought would mean a rejection of the hypothesis that the two analyses are consistent (i.e., if uncertainty is not sufficient to explain the difference between the theories, then the theories are distinguishable from one another). Instead, the authors claim that this is 'representing perfect agreement,' and I do not understand that statement. The bottom panel is a little better as at least the same trend is shown, but I am not sure I would classify the agreement as 'striking'. Overall, I think that the rest of the paper stands okay on its own without this figure and the related claims, so I would be satisfied if the flavor of the discussion about 4B were changed from 'striking confirmation' of their theory to something more along the lines of the results are 'not quite convincing enough evidence to reject their theory' and an appeal to other labs to collect more evidence to test it more completely.

Minor Comments

(1) Eqn 2 is unnecessarily confusing. It might be more clear if the numerator in the first term were: "gene expression with $R > 0$" and the denominator were "gene expression with $R = 0$", and make the right side: $\frac{p(R)}{p(0)}$.

(2) For Eqn (2) and (3) for the fold change, it would be helpful if it were written in a format that made it more obvious that this formula is for a function that depends on of the repressor concentration R, via the term ΔF_R. Maybe give it a name or symbol like $FC(R)$ or $\mathcal{F}(R)$ or whatever seems appropriate. Also, right after this

equation, it would be helpful if the text could explain which terms depend on R (e.g., ΔF_R) and which do not (e.g., ρ).

(3) Can you provide a reference for the statement, "Higher copy number TFs tend to have many binding sites over the genome, which should serve to pull them out of circulation and keep their effective association rates from rising too large." This seems logical to me, but it would be nice to know where I could look in the literature for confirmation.

(4) I found that much of the mathematics used for the calculation of means and variances in the supplemental sections to be unnecessarily complex. This is fine for the supplemental sections, but the authors may be interested in a much easier way to solve for these. In particular, the means can be found by solving a simple linear algebraic equation, and the variances can be found by solving a simple algebraic Lyapunov equation. See for example, the expressions about 10 lines down on the left column of page 1026 of the following paper:

M Khammash, Stochastic Gene Expression: Modeling, Analysis, and Identification, IFAC Proceedings Volumes, 2009

Philosophical Comments

The following are general comments to the authors that do not need to be addressed in their revisions.

- **Regarding differences in approaches**. Although I do not think this line of discussion is strictly relevant to my review, I do not understand the authors' response claiming that there is a fundamental philosophical difference between their approach and those we published in [Munsky, Mol. Syst. Biol.], [Neuert, Science, 2013], [Munsky, PNAS, 2018] or any of our other work on fitting and predicting single-cell gene regulation phenomena. Both approaches consider a continuous time jump Markov process with a discrete set of transcription factor binding sites and where the transcription activity is dependent on the current state of the promoter. The importance difference is not the number of models considered, but rather that the models from the Neuert/Munsky papers allowed for N different states with different positions at which the activator engages with the promoter and for time varying (i.e., non-steady state) levels for both the transcription activator concentrations and for the resulting mRNA distributions. (The reason for the more complex models with multiple states and different locations of action is exactly the same as that added to the current paper - to account for the potentially continuous nature of DNA conformational changes and how these changes interact with transcription factors). Similar to the authors' manuscript, the Neuert/Munsky papers solve for the means, variances, and distributions in different conditions and compare these solutions to data using maximum likelihood computations and Bayesian sampling, and they observe that the means are insufficient (even in the case of non-equilibrium data). Both groups of papers begin by posing one or several models that are based on the mechanistic knowledge of the system and justified by experimental data for

the mean expression levels and then attempt to discard models that are invalidated by the data variability. Both groups try to discard models that are overfit given the available data. Both groups have attempted to validate selected models by predicting orthogonal data (Munsky/Neuert predict responses to different genetic or environmental perturbations and the Phillips group seeks to predict parameters that could be measured using a different experimental technique).

- **Regarding 'Predictions'**. In the manuscript the term 'predict' appears to be used synonymously with capability to be 'refit' or 'reproduce' many alternate data sets. For example, I interpreted the statement, 'then we should be able to find values for the model parameters that can match theoretical predictions with single-molecule mRNA count distributions,' to mean that authors should be able to fit their model to data. In their manuscript, the authors do an excellent job to show that their models can be fit to a lot of different data sets, suggesting that such data alone may never be sufficient to invalidate their model. I think that is very impressive and important, but I would not call these a 'prediction'. Rather, I would say that the model is consistent with all the data or that it cannot be invalidated by the available experiments. The only counterexample is in Figure 4B where parameters are estimated using the model in one context and then compared to the same parameters as estimated using a different model in a different experimental context. Unfortunately, the analysis of those results is not as satisfying to me as it is to the authors.
- **Regarding citations**. To be perfectly honest, it was very difficult for me to discern what was the overall point of the first version of this manuscript and how it was meant to fit in the scope of the broader research landscape - thus the annoyance that was evident in my first report. This is better now, so most of my objection is overcome.

  As a rule of thumb, I think it appropriate to reference papers that are related to the work of interest and that share common themes. In this case, I expected the authors to reference key papers in the field that share the theme of the importance of carefully considering single-cell data (including the variability of that data) when attempting to identify mechanistic models of gene regulation – each of the authors in my previous laundry list did exactly that in one or more of their papers. Such referencing serves two goals: (1) it makes the reader better understand the relevance and motivation for your work, and (2) it makes your paper easier to find by the people who are most likely to benefit from reading the paper.
