# Supplementary material for: Reconciling kinetic and thermodynamic models of bacterial transcription
Source: PLoS Comput Biol. 2021 Jan 19;17(1):e1008572. doi: 10.1371/journal.pcbi.1008572 (PMC7845990; doi:10.1371/journal.pcbi.1008572)
Supplement: Attachment — Submitted filename: Review.docx [file pcbi.1008572.s003.docx]

Summary:

The authors have investigated the steady-state properties of several elementary gene expression models. A Bayesian framework was used to challenge the models with experimental data. The models were assessed according to (1) their likelihood given the data and (2) the posterior distribution of parameter values. They identified models involving transcriptional bursts as ones that explain the data without introducing poorly determined parameters. They applied the framework to show that transcription factor association and dissociation rates obtained using single-cell expression data are consistent with equilibrium constants obtained using mean population-level data.

Content:

The manuscript is organized into the following sections: 1. Introduction; 2. Mean Gene Expression; 3. Beyond Means in Gene Expression; 4. Finding the “right” model: Bayesian parameter inference; 5. Discussion and future work. There is an additional section 6. Methods, which consists of a link to a github repository. Sections 2 and 3 explore the behaviors of the models and to provide some justification for why a bursty transcription model might be suitable for the present study. Section 4 presents the results of assessing the models and identifying parameter values.

To review section 2, it is helpful to refer to the authors’ earlier work in Refs. 32 and 35. In Ref. 32, flow cytometry measurements of single-cell expression of a YFP reporter were obtained from 18 E. coli strains. In each strain, YFP expression was controlled by one of three lac operators (O1, O2, or O3) and one of six different LacI copy numbers. In Ref. 35, the data from Ref. 32 were considered and additional measurements were obtained from lacI point mutants. In these studies, an equation for the fold-change in expression was derived as a function of inducer concentration, repressor copy number, LacI-operator sequence binding energy, the energy difference between the active and inactive states of LacI, and the inducer dissociation constants in the active and inactive states of LacI. The model satisfactorily explained all of the data, as illustrated by collapsing all of the measurements onto a sigmoidal curve where the model is used to compute a new x-axis from the original inputs.

Section 2 essentially asks whether the results of Refs. 32 and 35 would still hold if the model of the promoter were to change. Five models were considered, in which the details of the promoter differ but in which repressor binding still only occurs through a single state. All of these models yield a similar form for the fold-change in expression, with the caveat that the x-axis needs to be shifted by a constant (that is, constant with respect to the variations introduced).

To review sections 3 and 4, it is helpful to refer additionally to the authors’ earlier work in Ref. 36 in which single-molecule mRNA FISH measurements of single-cell expression from a number of synthetic promoters were obtained. Constitutive promoter expression was modeled using a Poisson process, and the super-Poisson noise in expression from 18 constitutive promoters, as characterized using the Fano factor > 1, was attributed to quantization error, RNAP copy number variations, and gene copy number variations. Two promoters were placed under control of LacI, which in turn was expressed from an inducible promoter. The dependence of the Fano factor on LacI was measured and the elevation compared to the constitutive expression was explained using a simple model of repression.

Section 3 essentially asks whether the super-Poisson behavior (Fano factor > 1) of the constitutive promoters in Ref. 36 can be explained using an alternative promoter model instead of quantization error, RNAP copy number variations, and gene copy number variations. Of the five models considered, the two bursting models – one coarse-grained and one finer-grained – are the only ones that yield a Fano factor > 1. Here the authors jump ahead a bit, expressing a preference for the coarse-grained model – model 5 – based on Bayesian analysis not yet described. The finer-grained model – model 4 – was ruled out as having parameters that could not be identified.

Finally, section 4 applies the Bayesian framework to analyze the constitutive and repressor-regulated promoter models using data from Ref. 36. The authors immediately rule out the first three constitutive promoter models as having Fano factor <= 1. They cite unpublished Ref. 42 to rule out model 4, the finer-grained model, as having parameters that could not be identified using the data. They begin with model 1 as an example to gain intuition, and then move on to analyze model 5, and identify model parameters for 18 constitutive promoters. They find that, given some reasonable assumptions, the repressor binding parameters are consistent with prior equilibrium parameters obtained in refs. 32 and 33, and kinetic rates obtained in ref. 56.

These findings will be of interest to anyone who is interested in quantitative modeling of gene regulation; however issues with the presentation must be addressed before publication as a research article.

Presentation:

Here I insert a quote from the paper:

*In reflecting on these 5 models, the reader may feel that exploring a multitude of potential*

*models just to return to a very minimal phenomenological model of bursty transcription may*

*seem highly pedantic.*

The authors have hit on an important point here – it is possible to present sections 2-3 much more directly and concisely. In addition, and much more importantly, 2/3 the way into the paper, the reader still does not have the context necessary to identify the main results of the work and to understand their significance. Readers who are not familiar with the authors’ previous papers will be lost, and others will be perplexed about how this work connects.

The presentation would be improved substantially by eliminating most of sections 2-3 and refocusing and extending the introduction to motivate the present study in the context of the directly relevant literature rather than in the search for a model that unifies everything since Jacob and Monod. In setting the reader’s expectations for the latter, this paper will draw criticism for the limited possibilities explored, and for a narrow focus on the literature. For example, the paper does not really consider the possibilities of bimodal distributions or challenging the dynamical models with time-resolved experimental data. Above I sketched some ideas for how to draw a thread through this study, and the authors might have different ideas. The main thing is to help readers to understand precisely how the present work builds on and extends previous work. The discussion would be the place for the authors to speculate on the implications of their work for achieving larger goals, after the reader knows what they have done.

For example, in section 2, the essential result is that the coarse-grained bursty model (model 5 in Fig. 1C) yields the same fold-change expression as the elementary promoter model (model 1 in Fig. 1C), so that switching to this model doesn’t change the meaning of parameters obtained, e.g., in refs. 32 and 35. This point could be made without any math, simply by noting that both models 1 and 5 are equivalent from the perspective of repressor binding (both have only a single repressor-free state). Models 2, 3, and 4 don’t need to be considered at all, and the same goes for the models in Fig. 1B; insofar as the authors need to refer to them in this paper, they can cite the literature.

Similarly, in section 3, only models 1 and 5 need to be considered. Whereas the authors’ prior work in Ref. 36 using model 1 attributed the super-Poisson noise to extrinsic effects, here they bring the super-Poisson behavior into the model itself. The authors really only need to cite prior work describing how transcriptional bursting leads to a Fano factor > 1, and to note that whereas elsewhere the bursting model is applied to describe regulated expression (e.g. Munsky et al 2012, <https://science.sciencemag.org/content/336/6078/183.full>), here it is used to describe constitutive expression from the promoters in Ref. 36 (citing other work where a similar approach was taken, if there is any).

I offer the above ideas merely as suggestions of an approach to address the presentation issues. The authors might come up with a different approach; however, I must stress here that that the only way I could make sense of what was going on in this paper was to go back to the literature for the context, to ignore much of the exposition in the manuscript (which reads much more like a monograph from an intelligent and curious mind than a research paper), and to focus on the figures and the corresponding explanatory text. Fixing this problem will be essential if the important parts of this paper are to reach the intended audience.

Some details in Section 4 are missing. For example, there are no results to back up the conclusion that the parameters in model 4 cannot be identified. In addition, the Oid, O1, and O2 subscripts are introduced without information about what they correspond to; they are described in Figure 4 as operators, but some readers still won’t know what this means. This key section should be revised with an eye towards providing the reader with all of the evidence required to back up the conclusions, and defining all of the elements of the repression model.

In addition, the paper currently intermixes methods, results, and discussion in sections 2-4, with additional discussion in section 5. The methods should be broken out into a separate section, to follow the journal guidelines (there is no methods content in section 6 right now). The clarity of the exposition of the results would be improved by moving most of the interpretive comments and caveats to the discussion section or the supplementary material, as appropriate.

Some specific comments on the current manuscript follow. Some of these might refer to material that would be better left out of a revision, as described above.

p. 3, para 2. It would be good to walk the reader through the other panels of Fig. 1 in order here. As written Fig. 1B remains unaddressed, and Figs. 1C and 1D are mentioned but not explained.

p. 3, para 2. “…the same underlying functional form…” \Delta F is undefined in the equation. Please define in the Fig. 1 caption or here.

p. 3, Section 2. The main result of this section is that all of the models considered yield a similar form for the fold-change in expression, with the x-axis is shifted by a constant log \rho (that is, constant with respect to the variations introduced). Instead of walking through all of the derivations, it is possible to argue logically instead, observing the structure of the models in Fig. 1. The repressor-bound state (call it state 1) can only be reached from a single repressor-free state (call it state 0). The differences in the models are associated with the additional states of the repressor-free promoter, which draw density from states 0 and 1. Because the population ratio of states 0:1 must be the same in all of the models, the population ratio of (all repressor-free states):1 is greater than or equal to the 0:1 ratio. The difference in the log ratio is equal to log \rho. Model 5, like model 1, only consists of a single repressor-free state, which is why \rho = 1 for both of these models; values of the equilibrium constant obtained using models 1 and 5 therefore should be comparable. The authors might be able to find something to cite about this type of argument in a textbook or elsewhere in the literature.

p. 3, below Eq. 1. “…the models differ along several key axes: what states to consider and how to compute the probabilities of those states” In what sense are these axes? Or do the authors instead mean that the models differ in key aspects/characteristics.

p. 3, para 4. “As seen in Figure 1(B), even within the class of thermodynamic models…” Either use a consistent terminology equilibrium/thermodynamic or explain somewhere that they are being used to describe the same thing.

p. 5, para 2. “The second equality in Eq. 2 follows from assuming that the translation efficiency, i.e., the number of proteins translated per mRNA, is the same in both conditions.” It looks like there is no assumption about translation in the equation. Where does the assumption enter?

p. 5, last para. “…\rho which subsumes all details of transcription in the absence of repressors.” The meaning of \rho isn’t so clear yet. Can the authors try to explain it to the reader earlier, beyond the fact that it subsumes the details of transcription? Also aren’t the authors assuming that \rho captures the details of transcription both in the presence and absence of repressor?

p. 6, Eq. (3). The variables in this equation should be described immediately afterwards.

p. 9, para 3. “In fact, we suspect any model in which transcription proceeds through a multistep cycle must necessarily have \nu < 1.” See, e.g., Floyd et al (2010) Biophys J 99:360 (<https://www.ncbi.nlm.nih.gov/pmc/articles/PMC2905077/>) and refs therein.

p. 21, para 2. See also Senecal et al, 2104 (<https://www.sciencedirect.com/science/article/pii/S2211124714004471?via%3Dihub>, doi:10.1016/j.celrep.2014.05.053) and Friedman et al, 2006 (https://doi.org/10.1103/PhysRevLett.97.168302).

p. 26, Eq. (52). See Paulsson and Ehrenberg, 2000 (<https://journals.aps.org/prl/pdf/10.1103/PhysRevLett.84.5447>). Also see Friedman et al, 2006 (<https://doi.org/10.1103/PhysRevLett.97.168302>) which derives a continuous limit.

The data for the publication have been uploaded to github at <https://github.com/RPGroup-PBoC/bursty_transcription>. As it stands it’s not clear how to reproduce the results in the paper using what’s provided. There is code in the repo but the documentation could be improved, e.g., to indicate which scripts do what, and to indicate external dependencies.
